# Supplementary material for: Natural enemies of armored scales (Hemiptera: Diaspididae) and soft scales (Hemiptera: Coccidae) in Chile: Molecular and morphological identification
Source: PLoS One. 2019 Mar 18;14(3):e0205475. doi: 10.1371/journal.pone.0205475 (PMC6422274; doi:10.1371/journal.pone.0205475)
Supplement: S1 Table — (PDF) [file pone.0205475.s001.pdf]

Species name	Molecular Code	Sample Date	Region	Municipality	Latitud	Longitud	Host plant	Altitude	Family Host	Species Host	GenBank Access COI	GenBank Access 28S
Ablerus platensis	23938	17/04/2015	V	Quillota	32°50'54.81	71°13'57.89	*Citrus aurantifolia*	158	Diaspididae	*Lepidosaphes beckii*	-	MH455873
Ablerus platensis	23939	17/04/2015	V	Quillota	32°50'54.81	71°13'57.89	*Citrus aurantifolia*	158	Diaspididae	*Lepidosaphes beckii*	-	MH455874
Ablerus platensis	23945	17/04/2015	V	Quillota	32°50'54.81	71°13'57.89	*Citrus aurantifolia*	158	Diaspididae	*Lepidosaphes beckii*	-	MH455872
Ablerus platensis	23946	17/04/2015	V	Quillota	32°50'54.81	71°13'57.89	*Citrus aurantifolia*	158	Diaspididae	*Lepidosaphes beckii*	-	MH455875
Aphelinidae	23962	02/04/2015	VII	Pencahue	35°26'16.16	71°49'12.11	*Olea europaea*	72	Diaspididae	*Aspidiotus nerii*	MH456580	MH455710
Aphelinidae	23781	02/06/2015	IV	Ovalle	30°32'16.49	71°08'40.31	*Olea europaea*	286	Diaspididae	*Aspidiotus nerii*	-	MH455708
Aphelinidae	23912	15/04/2015	V	Quillota	32°53'46.38	71°12'33.68	*Hedera helix*	140	Coccidae	*Protopulvinaria pyriformis*	-	MH455709
Aphelinidae	24939	17/02/2016	IV	La Serena	29°56'22.49	71°08'50.13	*Olea europaea*	120	Diaspididae	*Saissetia coffeae*	-	MH455711
Aphelinidae	25063	03/12/2015	V	Hijuelas	32°51'43.41	71°03'57.2	*Citrus limon*	425	Diaspididae	*Aspidiotus nerii*	-	MH455712
Aphytis chilensis	23699	28/05/2015	VIII	San Carlos	36°26'04.58	71°57'45.18	*Hedera helix*	185	Diaspididae	*Aspidiotus nerii*	MH456432	-
Aphytis chilensis	23720	28/05/2015	VIII	San Carlos	36°26'04.58	71°57'45.18	*Hedera helix*	185	Diaspididae	*Aspidiotus nerii*	MH456433	-
Aphytis chilensis	23919	14/05/2015	Met	Paine	33°49'55.5	70°48'08.58	*Prunus amygdalus*	368	Diaspididae	*Diaspidiotus perniciosus*	MH456434	-
Aphytis chilensis	24808	12/10/2015	VI	Rancagua	34°10'01.28	70°44'01.55	*Hedera helix*	508	Coccidae	*Protopulvinaria pyriformis*	MH456435	MH455691
Aphytis chilensis	24809	12/10/2015	VI	Rancagua	34°10'01.28	70°44'01.55	*Hedera helix*	508	Coccidae	*Protopulvinaria pyriformis*	MH456436	MH455692
Aphytis chilensis	24846	07/10/2015	VIII	San Carlos	36°26'05.37	71°57'45.11	*Hedera helix*	185	Diaspididae	*Aspidiotus nerii*	MH456437	MH455694
Aphytis chilensis	24878	07/10/2015	VIII	San Carlos	36°26'03.47	71°57'45.46	*Ilex* sp.	184	Diaspididae	*Aspidiotus nerii*	MH456438	MH455695
Aphytis chilensis	24879	07/10/2015	VIII	San Carlos	36°26'03.47	71°57'45.46	*Ilex* sp.	184	Diaspididae	*Aspidiotus nerii*	MH456439	MH455696
Aphytis chilensis	24904	16/02/2016	IV	Coquimbo	29°59'05.32	71°15'00.2	*Citrus sinensis*	120	Diaspididae	*Lepidosaphes beckii*	MH456440	MH455698
Aphytis chilensis	27787	05/01/2016	VIII	San Carlos	36°26'05.37	71°57'44.98	*Hedera helix*	185	Diaspididae	*Aspidiotus nerii*	MH456441	MH455700
Aphytis chilensis	27788	05/01/2016	VIII	San Carlos	36°26'05.37	71°57'44.98	*Hedera helix*	185	Diaspididae	*Aspidiotus nerii*	MH456442	MH455701
Aphytis chilensis	27792	05/01/2016	VIII	San Carlos	36°26'05.37	71°57'44.98	*Hedera helix*	185	Diaspididae	*Aspidiotus nerii*	MH456443	MH455702
Aphytis chilensis	27793	05/01/2016	VIII	San Carlos	36°26'03.53	71°57'45.15	*Ilex* sp.	184	Diaspididae	*Aspidiotus nerii*	MH456444	MH455703
Aphytis chilensis	27795	05/01/2016	VI	Santa Cruz	34°38'20.76	71°21'52.13	*Hedera helix*	173	Diaspididae	*Aspidiotus nerii*	MH456445	MH455704
Aphytis chilensis	27796	05/01/2016	VI	Santa Cruz	34°38'20.76	71°21'52.13	*Hedera helix*	173	Diaspididae	*Aspidiotus nerii*	MH456446	MH455705
Aphytis chilensis	27825	05/01/2016	VI	Chimbarongo	34°38'33.1	70°59'20.22	*Hedera helix*	374	Diaspididae	-	MH456447	MH455706
Aphytis chilensis	23853	28/05/2015	VIII	San Carlos	36°26'04.58	71°57'45.18	*Ilex* sp.	185	Diaspididae	*Aspidiotus nerii*	-	MH455688
Aphytis chilensis	23854	28/05/2015	VIII	San Carlos	36°26'04.58	71°57'45.18	*Ilex* sp.	185	Diaspididae	*Aspidiotus nerii*	-	MH455689
Aphytis chilensis	23984	28/05/2015	VIII	San Carlos	36°26'04.58	71°57'45.18	*Hedera helix*	185	Diaspididae	*Aspidiotus nerii*	-	MH455690
Aphytis chilensis	24845	07/10/2015	VIII	San Carlos	36°26'05.37	71°57'45.11	*Hedera helix*	185	Diaspididae	*Aspidiotus nerii*	-	MH455693
Aphytis chilensis	24903	16/02/2016	IV	Coquimbo	29°59'05.32	71°15'00.2	*Citrus sinensis*	120	Diaspididae	*Lepidosaphes beckii*	-	MH455697
Aphytis chilensis	25116	03/12/2015	V	Hijuelas	32°51'55.64	71°04'01.6	*Cryptocarya alba*	496	Diaspididae	*Protopulvinaria pyriformis*	-	MH455699
Aphytis chilensis	27924	16/02/2016	IV	Coquimbo	29°59'05.32	71°15'00.2	*Citrus sinensis*	120	Diaspididae	*Aspidiotus nerii*	-	MH455707
Aphytis lepidosaphes	23695	02/06/2015	IV	Ovalle	30°32'16.49	71°08'40.31	*Citrus limon*	286	Diaspididae	*Lepidosaphes beckii*	-	MH455648
Aphytis lepidosaphes	23696	02/06/2015	IV	Ovalle	30°32'16.49	71°08'40.31	*Citrus limon*	286	Diaspididae	*Lepidosaphes beckii*	MH456647	MH455649
Aphytis lepidosaphes	23800	02/06/2015	IV	Ovalle	30°32'16.49	71°08'40.31	*Citrus limon*	286	Diaspididae	*Aspidiotus nerii*	MH456648	MH455650
Aphytis lepidosaphes	23801	02/06/2015	IV	Ovalle	30°32'16.49	71°08'40.31	*Citrus limon*	286	Diaspididae	*Aspidiotus nerii*	MH456649	MH455651
Aphytis lepidosaphes	23873	02/06/2015	IV	Ovalle	30°32'16.49	71°08'40.31	*Citrus limon*	286	Diaspididae	*Lepidosaphes beckii*	MH456650	MH455652
Aphytis lepidosaphes	23874	02/06/2015	IV	Ovalle	30°32'16.49	71°08'40.31	*Citrus limon*	286	Diaspididae	*Lepidosaphes beckii*	MH456651	MH455653
Aphytis lepidosaphes	23923	22/04/2015	V	Quillota	32°52'24.58	71°11'45.05	*Citrus sinensis*	158	Coccidae	*Lepidosaphes beckii*	MH456652	MH455654
Aphytis lepidosaphes	23924	22/04/2015	V	Quillota	32°52'24.58	71°11'45.05	*Citrus sinensis*	158	Coccidae	*Lepidosaphes beckii*	MH456653	MH455655
Aphytis lepidosaphes	23927	-	-	-	-	-	-	-	-	-	MH456654	MH455656
Aphytis lepidosaphes	23928	26/03/2015	VI	Placilla	34°37'30.91	71°07'27.14	*Citrus sinensis*	254	Diaspididae	*Lepidosaphes beckii*	MH456655	MH455657
Aphytis lepidosaphes	23953	17/04/2015	V	Quillota	32°50'54.81	71°13'57.89	*Citrus aurantifolia*	158	Diaspididae	*Lepidosaphes beckii*	MH456656	MH455647
Aphytis lepidosaphes	23954	17/04/2015	V	Quillota	32°50'54.81	71°13'57.89	*Citrus aurantifolia*	158	Diaspididae	*Lepidosaphes beckii*	MH456657	MH455658
Aphytis lepidosaphes	23960	17/04/2015	V	Quillota	32°50'54.81	71°13'57.89	*Citrus aurantifolia*	158	Diaspididae	*Lepidosaphes beckii*	MH456658	MH455659
Aphytis lepidosaphes	23961	17/04/2015	V	Quillota	32°50'54.81	71°13'57.89	*Citrus aurantifolia*	158	Diaspididae	*Lepidosaphes beckii*	MH456659	MH455660
Aphytis lepidosaphes	23691	28/05/2015	VII	Curico	34°58'40.96	71°12'24.27	*Nerium oleander*	234	Diaspididae	*Hemiberlesia rapax*	MH456646	-
Aphytis lepidosaphes	24920	05/01/2016	VI	Placilla	34°37'30.80	71°07'27.25	*Citrus sinensis*	254	Diaspididae	*Lepidosaphes beckii*	MH456666	-
Aphytis lepidosaphes	24005	21/02/2015	V	Valparaiso	33°02'51.00	71°37'50.00	*Citrus limon*	114	Diaspididae	*Lepidosaphes beckii*	MH456660	MH455662
Aphytis lepidosaphes	24887	16/02/2016	III	Vallenar	28°34'51.69	70°47'49.57	*Citrus sinensis*	470	Diaspididae	*Lepidosaphes beckii*	MH456661	MH455663
Aphytis lepidosaphes	24888	16/02/2016	III	Vallenar	28°34'51.69	70°47'49.57	*Citrus sinensis*	470	Diaspididae	*Lepidosaphes beckii*	MH456662	MH455664
Aphytis lepidosaphes	24917	16/02/2016	IV	Coquimbo	29°59'05.32	71°15'00.2	*Citrus sinensis*	120	Diaspididae	*Lepidosaphes beckii*	MH456663	MH455666
Aphytis lepidosaphes	24918	16/02/2016	IV	Coquimbo	29°59'05.32	71°15'00.2	*Citrus sinensis*	120	Diaspididae	*Lepidosaphes beckii*	MH456664	MH455667
Aphytis lepidosaphes	24919	05/01/2016	VI	Placilla	34°37'30.80	71°07'27.25	*Citrus sinensis*	254	Diaspididae	*Lepidosaphes beckii*	MH456665	MH455668

Species name	Molecular Code	Sample Date	Region	Municipality	Latitud	Longitud	Host plant	Altitude	Family Host	Species Host	GenBank Access COI	GenBank Access 28S
Aphytis lepidosaphes	24929	16/02/2016	IV	Coquimbo	29°59'05.32	71°15'00.2	*Citrus sinensis*	120	Diaspididae	*Lepidosaphes beckii*	MH456667	MH455669
Aphytis lepidosaphes	24930	16/02/2016	IV	Coquimbo	29°59'05.32	71°15'00.2	*Citrus sinensis*	120	Diaspididae	*Lepidosaphes beckii*	MH456668	MH455670
Aphytis lepidosaphes	25023	16/02/2016	III	Vallenar	28°34'43.53	70°47'42.21	*Olea europaea*	452	Coccidae	*Saissetia coffeae*	MH456669	MH455671
Aphytis lepidosaphes	25024	16/02/2016	III	Vallenar	28°34'51.69	70°47'49.57	*Citrus sinensis*	470	Diaspididae	*Lepidosaphes beckii*	MH456670	MH455672
Aphytis lepidosaphes	27807	07/01/2016	VII	Curico	34°59'01.09	71°13'17.73	*Citrus sinensis*	223	Coccidae	*Saissetia oleae*	MH456671	MH455673
Aphytis lepidosaphes	27809	07/01/2016	VII	Curico	34°59'01.09	71°13'17.73	*Citrus sinensis*	223	Diaspididae	*Saissetia oleae*	MH456672	MH455675
Aphytis lepidosaphes	27810	07/01/2016	VII	Curico	34°59'01.09	71°13'17.73	*Citrus sinensis*	223	Diaspididae	*Saissetia oleae*	MH456673	MH455676
Aphytis lepidosaphes	23970	01/04/2015	V	Cabildo	32°26'33.36	71°06'03.3	*Persea americana*	172	Coccidae	*Saissetia oleae*	-	MH455661
Aphytis lepidosaphes	24899	06/01/2016	VII	Curico	34°58'40.76	71°12'25.47	*Nerium oleander*	234	Coccidae	*Saissetia oleae*	-	MH455677
Aphytis lepidosaphes	24911	06/01/2016	VI	San Fernando	34°30'26.87	70°55'19.97	*Schinus latifolius*	313	Coccidae	*Saissetia oleae*	-	MH455665
Aphytis lepidosaphes	27808	07/01/2016	VII	Curico	34°59'01.09	71°13'17.73	*Citrus sinensis*	223	Coccidae	*Saissetia oleae*	-	MH455674
Aphytis melinus	23741	02/06/2015	IV	Monte Patria	30°51'04.56	70°46'14.47	*Persea americana*	715	Diaspididae	*Hemiberlesia lataniae*	MH456559	MH455678
Aphytis melinus	23803	02/06/2015	IV	Ovalle	30°32'16.49	71°08'40.31	*Citrus limon*	286	Diaspididae	*Aspidiotus nerii*	MH456561	MH455679
Aphytis melinus	23757	02/06/2015	IV	Ovalle	30°32'16.49	71°08'40.31	*Citrus limon*	286	Diaspididae	*Lepidosaphes beckii*	MH456560	-
Aphytis melinus	24029	22/04/2015	V	Quillota	32°52'27.82	71°11'41.39	*Citrus sinensis*	160	Diaspididae	*Hemiberlesia rapax*	MH456562	MH455642
Aphytis melinus	24031	08/04/2015	V	La Cruz	32°51'15.07	71°11'59.30	-	146	Diaspididae	*Aspidiotus nerii*	MH456563	MH455680
Aphytis melinus	24032	08/04/2015	V	La Cruz	32°51'15.07	71°11'59.30	-	146	Diaspididae	*Aspidiotus nerii*	MH456564	MH455681
Aphytis melinus	24045	21/02/2015	V	Valparaiso	33°02'51	71°37'50	*Citrus limon*	114	Diaspididae	*Aonidiella aurantii*	MH456565	MH455687
Aphytis melinus	24046	21/02/2015	V	Valparaiso	33°02'51	71°37'50	*Citrus limon*	114	Diaspididae	*Aonidiella aurantii*	MH456566	MH455682
Aphytis melinus	24151	28/05/2015	VII	Parral	36°08'48.21	71°49'15.61	*Aristotelia chilensis*	184	Coccidae	*Parthenolecanium corni*	MH456567	MH456378
Aphytis melinus	24154	18/08/2015	V	La Cruz	32°49'40.88	71°13'37.89	-	176	-	-	MH456568	MH456374
Aphytis melinus	25075	03/12/2015	V	Hijuelas	32°51'43.41	71°03'57.2	*Citrus limon*	425	Diaspididae	*Aspidiotus nerii*	MH456569	MH455686
Aphytis melinus	27826	05/01/2016	VI	Chimbarongo	34°38'33.1	70°59'20.22	*Hedera helix*	374	Diaspididae	-	MH456570	MH455685
Aphytis melinus	24130	02/06/2015	IV	Ovalle	30°32'16.49	71°08'40.31	*Persea americana*	286	Diaspididae	*Aspidiotus nerii*	-	MH455683
Aphytis melinus	25074	03/12/2015	V	Hijuelas	32°51'43.41	71°03'57.2	*Citrus limon*	425	Diaspididae	*Aspidiotus nerii*	-	MH455684
Aphytis notialis	23738	02/06/2015	IV	Ovalle	30°32'16.49	71°08'40.31	*Olea europaea*	286	Diaspididae	*Aspidiotus nerii*	-	MH455724
Aphytis notialis	23739	02/06/2015	IV	Ovalle	30°32'16.49	71°08'40.31	*Olea europaea*	286	Diaspididae	*Aspidiotus nerii*	-	MH455725
Aphytis notialis	23780	02/06/2015	IV	Ovalle	30°32'16.49	71°08'40.31	*Olea europaea*	286	Diaspididae	*Aspidiotus nerii*	-	MH455726
Aphytis notialis	23802	02/06/2015	IV	Ovalle	30°32'16.49	71°08'40.31	*Citrus limon*	286	Diaspididae	*Aspidiotus nerii*	-	MH455727
Aphytis notialis	23804	02/06/2015	IV	Ovalle	30°32'16.49	71°08'40.31	*Citrus limon*	286	Diaspididae	*Aspidiotus nerii*	-	MH455739
Aphytis notialis	23857	28/05/2015	VIII	San Carlos	36°26'04.58	71°57'45.18	*Ilex* sp.	185	Diaspididae	*Aspidiotus nerii*	-	MH455738
Aphytis notialis	23858	28/05/2015	VIII	San Carlos	36°26'04.58	71°57'45.18	*Ilex* sp.	185	Diaspididae	*Aspidiotus nerii*	-	MH455728
Aphytis notialis	23957	01/04/2015	V	Cabildo	32°26'34.2	71°05'48.17	*Persea americana*	158	Diaspididae	*Hemiberlesia lataniae*	-	MH455718
Aphytis notialis	24006	30/01/2015	VI	Chimbarongo	34°38'34.02	70°59'19.22	*Hedera helix*	376	Diaspididae	-	-	MH455723
Aphytis notialis	24007	30/01/2015	VI	Chimbarongo	34°38'34.02	70°59'19.22	*Hedera helix*	376	Diaspididae	-	-	MH455729
Aphytis notialis	24052	08/04/2015	V	La Cruz	32°51'20.88	71°11'16.79	*Olea europaea*	195	Diaspididae	*Aspidiotus nerii*	-	MH455721
Aphytis notialis	24059	08/04/2015	V	La Cruz	32°51'20.88	71°11'16.79	*Olea europaea*	195	Diaspididae	*Aspidiotus nerii*	-	MH455730
Aphytis notialis	24118	02/06/2015	IV	Los vilos	31°54'55.33	71°29'20.67	*Peumus boldus*	70	Diaspididae	*Aspidiotus nerii*	-	MH455731
Aphytis notialis	24786	09/09/2015	V	Quillota	32°52'19.03	71°11'44.94	*Olea europaea*	166	Coccidae	*Saissetia oleae*	-	MH455732
Aphytis notialis	24787	09/09/2015	V	Quillota	32°52'19.03	71°11'44.94	*Olea europaea*	166	Coccidae	*Saissetia oleae*	-	MH455722
Aphytis notialis	24881	07/10/2015	VIII	San Carlos	36°26'05.37	71°57'45.11	*Hedera helix*	185	Diaspididae	*Aspidiotus nerii*	-	MH455733
Aphytis notialis	24882	07/10/2015	VIII	San Carlos	36°26'05.37	71°57'45.11	*Hedera helix*	185	Diaspididae	*Aspidiotus nerii*	-	MH455734
Aphytis notialis	24940	17/02/2016	IV	La Serena	29°56'22.49	71°08'50.13	*Olea europaea*	120	Diaspididae	*Saissetia coffeae*	-	MH455735
Aphytis notialis	25134	03/12/2015	V	Hijuelas	32°51'55.64	71°04'01.6	*Cryptocarya alba*	496	Diaspididae	*Protopulvinaria pyriformis*	-	MH455719
Aphytis notialis	25135	03/12/2015	V	Hijuelas	32°51'55.64	71°04'01.6	*Cryptocarya alba*	496	Diaspididae	*Protopulvinaria pyriformis*	-	MH455720
Aphytis notialis	25145	18/12/2015	V	Quillota	32°51'53.9	71°11'12.19	*Persea americana*	185	Diaspididae	*Hemiberlesia lataniae*	-	MH455736
Aphytis notialis	27786	05/01/2016	VIII	San Carlos	36°26'05.37	71°57'44.98	*Hedera helix*	185	Diaspididae	*Aspidiotus nerii*	-	MH455737
Aphytis sp.I	23869	14/05/2015	Met	Santiago	33°34'07.93	70°38'03.37	*Malus domestica*	623	Diaspididae	*Diaspidiotus perniciosus*	MH456575	MH455645
Aphytis sp.I	23905	16/04/2015	V	Hijuelas	32°51'46.23	71°03'48.26	*Quillaja saponaria*	475	Diaspididae	*Diaspis chilensis*	MH456753	MH455713
Aphytis sp.I	23906	16/04/2015	V	Hijuelas	32°51'46.23	71°03'48.26	*Quillaja saponaria*	475	Diaspididae	*Diaspis chilensis*	MH456752	MH455714
Aphytis sp.I	23994	02/06/2015	IV	Monte Patria	30°43'14.76	70°52'16.89	*Prunus persica*	563	Diaspididae	*Diaspidiotus perniciosus*	MH456764	MH455715
Aphytis sp.I	23995	02/06/2015	IV	Monte Patria	30°43'14.76	70°52'16.89	*Prunus persica*	563	Diaspididae	*Diaspidiotus perniciosus*	MH456581	MH455716
Aphytis sp.I	24126	02/06/2015	IV	Monte Patria	30°53'41.72	70°39'28.89	*Olea europaea*	1278	Diaspididae	-	-	MH455717
Aphytis sp.I	24127	02/06/2015	IV	Ovalle	30°32'16.49	71°08'40.31	*Prunus persica*	286	Diaspididae	*Lepidosaphes ulmi*	-	MH455643

Species name	Molecular Code	Sample Date	Region	Municipality	Latitud	Longitud	Host plant	Altitude	Family Host	Species Host	GenBank Access COI	GenBank Access 28S
Aphytis sp.I	25154	06/01/2016	V	Putaendo	32°38'26.91	70°43'08.73	*Olea europaea*	790	Diaspididae	*Aspidiotus nerii*	-	MH455646
Aphytis sp.II	23820	02/06/2015	IV	Monte Patria	30°51'04.56	70°46'14.47	*Persea americana*	715	Diaspididae	*Hemiberlesia lataniae*	-	MH455757
Aphytis sp.II	23782	02/06/2015	IV	Ovalle	30°32'16.49	71°08'40.31	*Olea europaea*	286	Diaspididae	*Aspidiotus nerii*	MH456713	MH455748
Aphytis sp.II	23783	02/06/2015	IV	Ovalle	30°32'16.49	71°08'40.31	*Olea europaea*	286	Diaspididae	*Aspidiotus nerii*	MH456714	MH455753
Aphytis sp.II	23784	02/06/2015	IV	Ovalle	30°32'16.49	71°08'40.31	*Olea europaea*	286	Diaspididae	*Aspidiotus nerii*	MH456715	MH455754
Aphytis sp.II	23824	14/05/2015	Met	Paine	33°49'59.49	70°47'46.81	*Cydonia oblonga*	372	Diaspididae	*Diaspidiotus perniciosus*	MH456716	MH455758
Aphytis sp.II	23915	14/05/2015	Met	Paine	33°49'59.49	70°47'46.81	*Cydonia oblonga*	372	Diaspididae	*Diaspidiotus perniciosus*	MH456717	MH455755
Aphytis sp.II	23916	14/05/2015	Met	Paine	33°49'59.49	70°47'46.81	*Cydonia oblonga*	372	Diaspididae	*Diaspidiotus perniciosus*	MH456718	MH455756
Aphytis sp.II	24842	09/09/2015	V	Quillota	32°51'45.95	71°11'23.69	*Persea americana*	172	Diaspididae	*Hemiberlesia lataniae*	MH456719	MH455749
Aphytis sp.II	24843	09/09/2015	V	Quillota	32°51'45.95	71°11'23.69	*Persea americana*	172	Diaspididae	*Hemiberlesia lataniae*	MH456720	MH455750
Aphytis sp.II	24994	05/01/2016	VI	Santa Cruz	34°39'11.4	71°20'37.38	*Olea europaea*	177	Coccidae	*Saissetia oleae*	MH456721	MH455751
Aphytis sp.II	24998	16/02/2016	III	Huasco	28°28'53.84	71°09'28.44	*Olea europaea*	37	Diaspididae	-	MH456722	MH455752
Aphytis sp.II	24106	26/03/2015	Met	San Bernando	33°40'09.98	70°43'20.9	*Euonymus* sp.	544	Diaspididae	*Aspidiotus nerii*	-	MH455740
Aphytis sp.II	24107	26/03/2015	Met	San Bernando	33°40'09.98	70°43'20.9	*Euonymus* sp.	544	Diaspididae	*Aspidiotus nerii*	-	MH455741
Aphytis sp.II	24116	02/06/2015	IV	Monte Patria	30°43'14.76	70°52'16.89	*Prunus persica*	563	Diaspididae	*Diaspidiotus perniciosus*	-	MH455742
Aphytis sp.II	24122	02/06/2015	IV	Monte Patria	30°51'04.56	70°46'14.47	*Persea americana*	715	Diaspididae	*Lepidosaphes ulmi*	-	MH455747
Aphytis sp.II	24861	-	-	-	-	-	-	-	-	-	-	MH455743
Aphytis sp.II	24862	-	-	-	-	-	-	-	-	-	-	MH455744
Aphytis sp.II	24956	16/02/2016	III	Vallenar	28°34'51.69	70°47'49.57	*Prunus cerasus*	470	Diaspididae	*Diaspidiotus perniciosus*	-	MH455745
Aphytis sp.II	27811	06/01/2016	VI	San Fernando	34°30'26.87	70°55'19.97	*Quillaja saponaria*	313	Diaspididae	*Diaspis chilensis*	-	MH455746
Chartocerus niger	23936	17/04/2015	V	Quillota	32°50'54.81	71°13'57.89	*Citrus aurantifolia*	158	Diaspididae	*Lepidosaphes beckii*	MH456579	MH455917
Coccophaginae sp.I	23826	14/05/2015	V	Casablanca	33°21'50.8	71°18'46.34	*Quillaja saponaria*	313	Diaspididae	*Melanaspis sitreana*	MH456744	MH455914
Coccophaginae sp.II	23949	17/04/2015	V	Quillota	32°50'54.81	71°13'57.89	*Citrus aurantifolia*	158	Diaspididae	*Lepidosaphes beckii*	MH456756	MH455764
Coccophaginae sp.II	23950	17/04/2015	V	Quillota	32°50'54.81	71°13'57.89	*Citrus aurantifolia*	158	Diaspididae	*Lepidosaphes beckii*	MH456757	MH455765
Coccophagus gurney	23947	01/04/2015	V	Cabildo	32°26'33.36	71°06'03.3	*Persea americana*	172	-	-	MH456689	MH455918
Coccophagus gurney	23948	01/04/2015	V	Cabildo	32°26'33.36	71°06'03.3	*Persea americana*	172	-	-	MH456690	MH455919
Coccophagus gurney	23963	01/04/2015	V	Cabildo	32°26'46.47	71°05'35.7	*Persea americana*	159	-	-	MH456691	MH455920
Coccophagus gurney	23978	28/05/2015	VIII	Chillan	36°35'35.85	72°04'18.56	*Choisya ternata*	148	-	-	MH456692	MH455921
Coccophagus gurney	23701	28/05/2015	VIII	Chillan	36°35'35.85	72°04'18.56	*Choisya ternata*	148	-	-	MH456688	-
Coccophagus gurney	24152	16/04/2015	V	Hijuelas	32°51'55.35	71°04'01.65	*Quillaja saponaria*	471	-	-	MH456693	MH456372
Coccophagus gurney	27817	05/01/2016	Met	Paine	33°51'19.8	70°45'21.38	*Hedera helix*	378	-	-	MH456694	MH455922
Coccophagus gurney	27830	05/01/2016	Met	Paine	33°51'19.8	70°45'21.38	*Hedera helix*	378	-	-	MH456695	MH455923
Coccophagus yoshidae	23822	28/05/2015	VIII	San Carlos	36°25'58.64	71°57'42.88	*Maytenus boaria*	181	Coccidae	*Saissetia oleae*	-	MH455783
Coccophagus yoshidae	23823	28/05/2015	VIII	San Carlos	36°25'58.64	71°57'42.88	*Maytenus boaria*	181	Coccidae	*Saissetia oleae*	-	MH455781
Coccophagus yoshidae	23697	28/05/2015	VIII	San Carlos	36°26'04.58	71°57'45.18	*Aristotelia chilensis*	185	Diaspididae	*Hemiberlesia rapax*	MH456402	-
Coccophagus yoshidae	23706	28/05/2015	VIII	San Carlos	36°25'58.64	71°57'42.88	*Maytenus boaria*	181	Coccidae	*Saissetia oleae*	MH456403	-
Coccophagus yoshidae	23708	28/05/2015	VII	Parral	36°08'57.53	71°49'16.5	*Nerium oleander*	182	Coccidae	*Saissetia oleae*	MH456404	-
Coccophagus yoshidae	24853	21/09/2015	V	Putaendo	32°37'21.80	70°43'00.14	*Citrus clementina*	831	Diaspididae	-	MH456414	-
Coccophagus yoshidae	24922	05/01/2016	VII	Parral	36°08'49.7	71°49'09.76	*Citrus limon*	178	Coccidae	*Saissetia oleae*	MH456420	-
Coccophagus yoshidae	23788	01/04/2015	V	Cabildo	32°26'22.3	71°05'49.72	*Persea americana*	173	Diaspididae	*Saissetia oleae*	MH456483	MH455782
Coccophagus yoshidae	23751	28/05/2015	VII	Curico	34°58'40.96	71°12'24.27	*Nerium oleander*	234	Coccidae	*Saissetia oleae*	MH456480	-
Coccophagus yoshidae	23772	06/05/2015	V	Casablanca	33°27'25.54	71°23'40.84	*Schinus latifolius*	241	Coccidae	*Saissetia oleae*	MH456481	-
Coccophagus yoshidae	23934	01/04/2015	V	Cabildo	32°26'33.36	71°06'03.3	*Persea americana*	172	Coccidae	*Saissetia oleae*	MH456489	-
Coccophagus yoshidae	24149	22/04/2015	V	Quillota	32°52'19.53	71°11'39.09	*Maytenus boaria*	165	Coccidae	*Saissetia oleae*	MH456502	-
Coccophagus yoshidae	24802	21/09/2015	V	Cabildo	32°28'23.98	71°07'07.61	*Olea europaea*	133	Diaspididae	*Saissetia oleae*	MH456507	-
Coccophagus yoshidae	23884	06/05/2015	V	Casablanca	33°27'25.54	71°23'40.84	*Schinus latifolius*	241	Coccidae	*Saissetia oleae*	MH456484	MH455784
Coccophagus yoshidae	23885	06/05/2015	V	Casablanca	33°27'25.54	71°23'40.84	*Schinus latifolius*	241	Coccidae	*Saissetia oleae*	MH456485	MH455785
Coccophagus yoshidae	23886	06/05/2015	V	Casablanca	33°27'25.54	71°23'40.84	*Schinus latifolius*	241	Coccidae	*Saissetia oleae*	MH456486	MH455786
Coccophagus yoshidae	23903	16/04/2015	V	Hijuelas	32°51'55.35	71°04'01.65	*Cryptocarya alba*	471	Coccidae	*Protopulvinaria pyriformis*	MH456487	MH455787
Coccophagus yoshidae	23904	16/04/2015	V	Hijuelas	32°51'55.35	71°04'01.65	*Cryptocarya alba*	471	Coccidae	*Protopulvinaria pyriformis*	MH456488	MH455788
Coccophagus yoshidae	23935	01/04/2015	V	Cabildo	32°26'33.36	71°06'03.3	*Persea americana*	172	Coccidae	*Saissetia oleae*	MH456490	MH455789
Coccophagus yoshidae	23943	01/04/2015	V	Cabildo	32°26'33.36	71°06'03.3	*Persea americana*	172	Coccidae	*Saissetia oleae*	MH456491	MH455790
Coccophagus yoshidae	23944	01/04/2015	V	Cabildo	32°26'33.36	71°06'03.3	*Persea americana*	172	Coccidae	*Saissetia oleae*	MH456492	MH455871
Coccophagus yoshidae	23985	28/05/2015	VII	Parral	36°08'57.53	71°49'16.5	*Nerium oleander*	182	Coccidae	*Saissetia oleae*	MH456405	MH455791

Species name	Molecular Code	Sample Date	Region	Municipality	Latitud	Longitud	Host plant	Altitude	Family Host	Species Host	GenBank Access COI	GenBank Access 28S
Coccophagus yoshidae	23986	28/05/2015	VII	Parral	36°08'57.53	71°49'16.5	*Nerium oleander*	182	Coccidae	*Saissetia oleae*	MH456406	MH455792
Coccophagus yoshidae	24039	08/04/2015	V	La Cruz	32°51'20.09	71°11'26.48	*Olea europaea*	176	Coccidae	*Saissetia oleae*	MH456494	MH455794
Coccophagus yoshidae	24042	08/04/2015	V	La Cruz	32°51'20.09	71°11'26.48	*Olea europaea*	176	Coccidae	*Saissetia oleae*	MH456495	MH455795
Coccophagus yoshidae	24043	08/04/2015	V	La Cruz	32°51'20.09	71°11'26.48	*Olea europaea*	176	Coccidae	*Saissetia oleae*	MH456496	MH455796
Coccophagus yoshidae	24044	08/04/2015	V	La Cruz	32°51'20.09	71°11'26.48	*Olea europaea*	176	Coccidae	*Saissetia oleae*	MH456497	MH455797
Coccophagus yoshidae	24047	08/04/2015	V	La Cruz	32°51'20.09	71°11'26.48	*Olea europaea*	176	Coccidae	*Saissetia oleae*	MH456498	MH455798
Coccophagus yoshidae	24048	08/04/2015	V	La Cruz	32°51'20.09	71°11'26.48	*Olea europaea*	176	Coccidae	*Saissetia oleae*	MH456499	MH455799
Coccophagus yoshidae	24063	16/04/2015	V	Hijuelas	32°51'55.35	71°04'01.65	*Schinus latifolius*	471	Coccidae	*Saissetia oleae*	MH456500	MH455802
Coccophagus yoshidae	24064	16/04/2015	V	Hijuelas	32°51'55.35	71°04'01.65	*Schinus latifolius*	471	Coccidae	*Saissetia oleae*	MH456501	MH455803
Coccophagus yoshidae	24150	08/04/2015	V	La Cruz	32°51'29.52	71°11'19.04	*Persea americana*	186	Diaspididae	*Protopulvinaria pyriformis*	MH456503	MH456371
Coccophagus yoshidae	24784	10/09/2015	V	Ocoa	32°51'36.49	71°04'47.59	*Persea americana*	329	Coccidae	*Saissetia oleae*	MH456504	MH455811
Coccophagus yoshidae	24785	10/09/2015	V	Ocoa	32°51'36.49	71°04'47.59	*Persea americana*	329	Coccidae	*Saissetia oleae*	MH456505	MH455812
Coccophagus yoshidae	24801	21/09/2015	V	Cabildo	32°28'23.98	71°07'07.61	*Olea europaea*	133	Diaspididae	*Saissetia oleae*	MH456506	MH455813
Coccophagus yoshidae	24816	20/09/2015	Met	Til Til	33°06'50.88	70°55'39.27	*Nerium oleander*	564	Coccidae	*Saissetia oleae*	MH456407	MH455814
Coccophagus yoshidae	24817	20/09/2015	Met	Til Til	33°06'50.88	70°55'39.27	*Nerium oleander*	564	Coccidae	*Saissetia oleae*	MH456408	MH455815
Coccophagus yoshidae	24847	12/10/2015	VI	Coya	34°12'23.5	70°31'50.83	*Citrus sinensis*	786	Coccidae	*Saissetia oleae*	MH456409	MH455780
Coccophagus yoshidae	24849	09/10/2015	VI	Rengo	34°21'22.75	70°50'45.48	*Nerium oleander*	338	Coccidae	*Saissetia oleae*	MH456410	MH455817
Coccophagus yoshidae	24850	09/10/2015	VI	Rengo	34°21'22.75	70°50'45.48	*Nerium oleander*	338	Coccidae	*Saissetia oleae*	MH456411	MH455818
Coccophagus yoshidae	24851	21/09/2015	V	Cabildo	32°26'34.40	71°05'52.94	Unknown	158	Coccidae	*Parthenolecanium corni*	MH456412	MH455819
Coccophagus yoshidae	24852	21/09/2015	V	Cabildo	32°26'34.40	71°05'52.94	Unknown	158	Coccidae	*Parthenolecanium corni*	MH456413	MH455820
Coccophagus yoshidae	24874	21/09/2015	V	Putaendo	32°37'21.80	70°43'00.14	*Citrus clementina*	831	Diaspididae	*Saissetia oleae*	MH456415	MH455823
Coccophagus yoshidae	24875	21/09/2015	V	Putaendo	32°37'21.80	70°43'00.14	*Citrus clementina*	831	Diaspididae	*Saissetia oleae*	MH456416	MH455824
Coccophagus yoshidae	24880	12/10/2015	VI	Coya	34°12'23.5	70°31'50.83	*Citrus sinensis*	786	Coccidae	*Saissetia oleae*	MH456417	MH455825
Coccophagus yoshidae	24912	06/01/2016	VII	Curico	34°58'40.76	71°12'25.47	*Nerium oleander*	234	Coccidae	*Saissetia oleae*	MH456418	MH455826
Coccophagus yoshidae	24921	05/01/2016	VII	Parral	36°08'49.7	71°49'09.76	*Citrus limon*	178	Coccidae	*Saissetia oleae*	MH456419	MH455827
Coccophagus yoshidae	24954	17/02/2016	IV	La Serena	29°56'22.49	71°08'50.13	*Olea europaea*	120	Coccidae	*Saissetia coffeae*	MH456421	MH455828
Coccophagus yoshidae	24955	17/02/2016	IV	La Serena	29°56'22.49	71°08'50.13	*Olea europaea*	120	Coccidae	*Saissetia coffeae*	MH456609	MH455829
Coccophagus yoshidae	25018	16/02/2016	IV	La Serena	29°55'08.61	71°14'33.31	*Citrus sinensis*	92	Coccidae	*Saissetia oleae*	MH456610	MH455832
Coccophagus yoshidae	25119	07/12/2015	Met	San José de Maipo	33°40'43.15	70°20'27.03	*Maytenus boaria*	1005	Coccidae	*Saissetia oleae*	MH456422	MH455851
Coccophagus yoshidae	27797	06/01/2016	V	Putaendo	32°38'26.91	70°43'08.73	*Olea europaea*	790	Coccidae	*Saissetia oleae*	MH456423	MH455858
Coccophagus yoshidae	27798	06/01/2016	V	Putaendo	32°38'26.91	70°43'08.73	*Olea europaea*	790	Coccidae	*Saissetia oleae*	MH456424	MH455859
Coccophagus yoshidae	27819	05/01/2016	Met	Paine	33°51'19.8	70°45'21.38	*Hedera helix*	378	Coccidae	*Protopulvinaria pyriformis*	MH456425	MH455861
Coccophagus yoshidae	27854	05/01/2016	Met	San Bernardo	33°40'11.39	70°43'19.6	*Nerium oleander*	542	Coccidae	*Saissetia oleae*	MH456426	MH455863
Coccophagus yoshidae	27855	05/01/2016	Met	San Bernardo	33°40'11.39	70°43'19.6	*Nerium oleander*	542	Coccidae	*Saissetia oleae*	MH456427	MH455864
Coccophagus yoshidae	27856	05/01/2016	Met	San Bernardo	33°40'11.39	70°43'19.6	*Nerium oleander*	542	Coccidae	*Saissetia oleae*	MH456428	MH455865
Coccophagus yoshidae	27857	05/01/2016	Met	San Bernardo	33°40'11.39	70°43'19.6	*Nerium oleander*	542	Coccidae	*Saissetia oleae*	MH456429	MH455866
Coccophagus yoshidae	27864	05/01/2016	Met	Paine	33°51'17.41	70°45'49.47	*Citrus limon*	375	Coccidae	*Saissetia oleae*	MH456430	MH455867
Coccophagus yoshidae	27865	05/01/2016	Met	Paine	33°51'17.41	70°45'49.47	*Citrus limon*	375	Coccidae	*Saissetia oleae*	MH456431	MH455868
Coccophagus yoshidae	24049	27/02/2015	V	Hijuelas	32°51'27.1	71°03'59.58	*Persea americana*	351	Coccidae	*Saissetia oleae*	-	MH455800
Coccophagus yoshidae	24050	27/02/2015	V	Hijuelas	32°51'27.1	71°03'59.58	*Persea americana*	351	Coccidae	*Saissetia oleae*	-	MH455801
Coccophagus yoshidae	24062	08/04/2015	V	La Cruz	32°51'15.99	71°11'04.56	*Persea americana*	214	Coccidae	*Saissetia oleae*	-	MH455870
Coccophagus yoshidae	24073	08/04/2015	V	La Cruz	32°51'15.99	71°11'04.56	*Persea americana*	214	Coccidae	*Saissetia oleae*	-	MH455804
Coccophagus yoshidae	24074	08/04/2015	V	La Cruz	32°51'15.99	71°11'04.56	*Persea americana*	214	Coccidae	*Saissetia oleae*	-	MH455805
Coccophagus yoshidae	24123	16/04/2015	V	Hijuelas	32°51'24.77	71°03'51.8	*Persea americana*	370	Coccidae	*Saissetia oleae*	-	MH455869
Coccophagus yoshidae	24124	16/04/2015	V	Hijuelas	32°51'24.77	71°03'51.8	*Persea americana*	370	Coccidae	*Saissetia oleae*	-	MH455806
Coccophagus yoshidae	24131	16/04/2015	V	Hijuelas	32°51'24.77	71°03'51.8	*Persea americana*	370	Coccidae	*Saissetia oleae*	-	MH455807
Coccophagus yoshidae	24132	16/04/2015	V	Hijuelas	32°51'24.77	71°03'51.8	*Persea americana*	370	Coccidae	*Saissetia oleae*	-	MH455808
Coccophagus yoshidae	24135	16/04/2015	V	Hijuelas	32°51'24.77	71°03'51.8	*Persea americana*	370	Coccidae	*Saissetia oleae*	-	MH455809
Coccophagus yoshidae	24136	16/04/2015	V	Hijuelas	32°51'24.77	71°03'51.8	*Persea americana*	370	Coccidae	*Saissetia oleae*	-	MH455810
Coccophagus yoshidae	24834	15/09/2015	V	Hijuelas	32°51'26.27	71°03'54.65	*Persea americana*	368	Coccidae	*Saissetia oleae*	-	MH455816
Coccophagus yoshidae	24858	15/09/2015	V	Hijuelas	32°51'30.92	71°03'48.03	Unknown	392	Coccidae	*Saissetia oleae*	-	MH455821
Coccophagus yoshidae	24859	15/09/2015	V	Hijuelas	32°51'30.92	71°03'48.03	Unknown	392	Coccidae	*Saissetia oleae*	-	MH455822
Coccophagus yoshidae	24980	05/01/2016	VI	Santa Cruz	34°39'11.4	71°20'37.38	*Olea europaea*	177	Coccidae	*Saissetia oleae*	-	MH455830
Coccophagus yoshidae	24981	17/02/2016	IV	La Serena	29°56'22.49	71°08'50.13	*Olea europaea*	120	Diaspididae	*Saissetia coffeae*	-	MH455831

Species name	Molecular Code	Sample Date	Region	Municipality	Latitud	Longitud	Host plant	Altitude	Family Host	Species Host	GenBank Access COI	GenBank Access 28S
Coccophagus yoshidae	25044	03/12/2015	V	Hijuelas	32°52'08.53	71°04'29.07	*Persea americana*	470	Coccidae	*Saissetia oleae*	-	MH455833
Coccophagus yoshidae	25045	03/12/2015	V	Hijuelas	32°52'08.53	71°04'29.07	*Persea americana*	470	Coccidae	*Saissetia oleae*	-	MH455834
Coccophagus yoshidae	25051	03/12/2015	V	Hijuelas	32°51'44.15	71°03'55.95	*Persea americana*	459	Coccidae	*Saissetia oleae*	-	MH455836
Coccophagus yoshidae	25052	03/12/2015	V	Hijuelas	32°51'44.15	71°03'55.95	*Persea americana*	459	Coccidae	*Saissetia oleae*	-	MH455837
Coccophagus yoshidae	25053	03/12/2015	V	Hijuelas	32°51'59.46	71°04'33.13	*Cryptocarya alba*	410	Coccidae	*Saissetia oleae*	-	MH455838
Coccophagus yoshidae	25054	03/12/2015	V	Hijuelas	32°51'59.46	71°04'33.13	*Cryptocarya alba*	410	Coccidae	*Saissetia oleae*	-	MH455839
Coccophagus yoshidae	25055	03/12/2015	V	Hijuelas	32°51'52.16	71°04'18.45	*Persea americana*	392	Coccidae	-	-	MH455840
Coccophagus yoshidae	25071	03/12/2015	V	Hijuelas	32°51'27.35	71°04'00.06	*Persea americana*	351	Coccidae	*Saissetia oleae*	-	MH455841
Coccophagus yoshidae	25072	03/12/2015	V	Hijuelas	32°51'27.35	71°04'00.06	*Persea americana*	351	Coccidae	*Saissetia oleae*	-	MH455842
Coccophagus yoshidae	25079	03/12/2015	V	Hijuelas	32°51'44.15	71°03'55.95	*Persea americana*	459	Coccidae	*Saissetia oleae*	-	MH455843
Coccophagus yoshidae	25080	03/12/2015	V	Hijuelas	32°51'44.15	71°03'55.95	*Persea americana*	459	Coccidae	*Saissetia oleae*	-	MH455844
Coccophagus yoshidae	25089	03/12/2015	V	Hijuelas	32°51'55.64	71°04'01.6	*Schinus latifolius*	496	Coccidae	*Saissetia oleae*	-	MH455845
Coccophagus yoshidae	25090	03/12/2015	V	Hijuelas	32°51'55.64	71°04'01.6	*Schinus latifolius*	496	Coccidae	*Saissetia oleae*	-	MH455846
Coccophagus yoshidae	25095	03/12/2015	V	Hijuelas	32°51'43.41	71°03'57.2	*Citrus limon*	425	Coccidae	*Saissetia oleae*	-	MH455847
Coccophagus yoshidae	25096	03/12/2015	V	Hijuelas	32°51'43.41	71°03'57.2	*Citrus limon*	425	Coccidae	*Saissetia oleae*	-	MH455848
Coccophagus yoshidae	25113	16/12/2015	V	Quillota	32°56'06.20	71°15'28	*Citrus limon*	230	Coccidae	*Ceroplastes sinensis*	-	MH455849
Coccophagus yoshidae	25114	16/12/2015	V	Quillota	32°56'06.20	71°15'28	*Citrus limon*	230	Coccidae	*Ceroplastes sinensis*	-	MH455850
Coccophagus yoshidae	25120	07/12/2015	Met	San José de Maipo	33°40'43.15	70°20'27.03	*Maytenus boaria*	1005	Coccidae	*Saissetia oleae*	-	MH455852
Coccophagus yoshidae	25129	16/12/2015	V	Quillota	32°56'06.20	71°15'28	*Citrus limon*	230	Coccidae	*Ceroplastes sinensis*	-	MH455853
Coccophagus yoshidae	25130	03/12/2015	V	Hijuelas	32°51'55.64	71°04'01.6	*Cryptocarya alba*	496	Coccidae	*Protopulvinaria pyriformis*	-	MH455854
Coccophagus yoshidae	25131	03/12/2015	V	Hijuelas	32°51'55.64	71°04'01.6	*Cryptocarya alba*	496	Coccidae	*Protopulvinaria pyriformis*	-	MH455855
Coccophagus yoshidae	25139	18/12/2015	V	Quillota	32°51'54.4	71°11'10.98	*Schinus latifolius*	185	Coccidae	*Saissetia oleae*	-	MH455856
Coccophagus yoshidae	25140	18/12/2015	V	Quillota	32°51'54.4	71°11'10.98	*Schinus latifolius*	185	Coccidae	*Saissetia oleae*	-	MH455857
Coccophagus yoshidae	27815	05/01/2016	VII	Parral	36°08'58.12	71°49'15.83	*Nerium oleander*	182	Coccidae	*Saissetia oleae*	-	MH455860
Coccophagus yoshidae	27843	06/01/2016	V	Catemu	32°47'28.56	70°52'11.09	*Nerium oleander*	499	Coccidae	*Saissetia oleae*	-	MH455862
Encarsia citrina	23817	02/06/2015	IV	Monte Patria	30°43'06.29	70°52'32.47	*Citrus* sp.	505	Coccidae	*Coccus hesperidum*	-	MH455767
Encarsia citrina	23913	14/05/2015	Met	Paine	33°49'59.49	70°47'46.81	*Cydonia oblonga*	372	Diaspididae	*Diaspidiotus perniciosus*	-	MH455768
Encarsia citrina	23914	14/05/2015	Met	Paine	33°49'59.49	70°47'46.81	*Cydonia oblonga*	372	Diaspididae	*Diaspidiotus perniciosus*	-	MH455769
Encarsia citrina	23931	02/04/2015	VII	Pencahue	35°26'16.16	71°49'12.11	*Olea europaea*	72	Diaspididae	*Aspidiotus nerii*	-	MH455770
Encarsia citrina	23932	02/04/2015	VII	Pencahue	35°26'16.16	71°49'12.11	*Olea europaea*	72	Diaspididae	*Aspidiotus nerii*	-	MH455771
Encarsia citrina	23959	01/04/2015	V	Cabildo	32°26'40.34	71°06'08.74	*Persea americana*	170	Diaspididae	*Hemiberlesia lataniae*	-	MH455772
Encarsia citrina	23980	28/05/2015	VIII	San Carlos	36°26'04.58	71°57'45.18	*Hedera helix*	185	Diaspididae	*Aspidiotus nerii*	-	MH455773
Encarsia citrina	23775	28/05/2015	VIII	San Carlos	36°26'04.58	71°57'45.18	*Hedera helix*	185	Diaspididae	*Aspidiotus nerii*	MH456482	MH455766
Encarsia citrina	24146	22/04/2015	VII	Pencahue	35°26'16.16	71°49'12.11	*Olea europaea*	72	Diaspididae	*Aspidiotus nerii*	-	MH455774
Encarsia citrina	24147	22/04/2015	VII	Pencahue	35°26'16.16	71°49'12.11	*Olea europaea*	72	Diaspididae	*Aspidiotus nerii*	-	MH455775
Encarsia citrina	24771	07/10/2015	VIII	San Carlos	36°26'05.37	71°57'45.11	*Hedera helix*	185	Diaspididae	*Aspidiotus nerii*	-	MH455776
Encarsia citrina	24772	07/10/2015	VIII	San Carlos	36°26'05.37	71°57'45.11	*Hedera helix*	185	Diaspididae	*Aspidiotus nerii*	-	MH455777
Encarsia citrina	25040	17/11/2015	XIV	Valdivia	39°48'22.55	73°15'03.7	Unknown	18	Diaspididae	*Aonidomytilus* sp.	-	MH455778
Encarsia citrina	25041	25/11/2015	X	Frutillar	41°08'08.03	73°01'37.14	*Ilex* sp.	58	Coccidae	*Aonidomytilus* sp.	-	MH455779
Encarsia hispida	23832	28/05/2015	VII	Parral	36°08'48.21	71°49'15.61	*Aristotelia chilensis*	184	-	-	MH456571	MH455908
Encarsia hispida	23833	28/05/2015	VII	Parral	36°08'48.21	71°49'15.61	*Aristotelia chilensis*	184	-	-	MH456572	MH455909
Encarsia hispida	24060	08/04/2015	V	La Cruz	32°51'20.88	71°11'16.79	*Olea europaea*	195	-	-	MH456574	-
Encarsia hispida	24051	08/04/2015	V	La Cruz	32°51'20.88	71°11'16.79	*Olea europaea*	195	-	-	MH456573	MH455910
Encarsia lounsburyi	23992	21/05/2015	IV	La Serena	29°54'58.14	71°14'43.09	*Hedera helix*	47	Coccidae	*Protopulvinaria pyriformis*	MH456759	MH455759
Encarsia lounsburyi	23993	21/05/2015	IV	La Serena	29°54'58.14	71°14'43.09	*Hedera helix*	47	Coccidae	*Protopulvinaria pyriformis*	MH456760	MH455760
Encarsia lounsburyi	24953	16/02/2016	IV	La Serena	29°55'08.61	71°14'33.31	*Hedera helix*	92	Coccidae	*Protopulvinaria pyriformis*	MH456761	MH455761
Encarsia lounsburyi	25013	16/02/2016	III	Vallenar	28°34'43.53	70°47'42.21	*Olea europaea*	452	Coccidae	*Saissetia coffeae*	MH456762	MH455762
Encarsia lounsburyi	25014	16/02/2016	III	Huasco	28°28'53.84	71°09'28.44	*Olea europaea*	37	Diaspididae	-	MH456763	MH455763
Encarsia sp.	25093	03/12/2015	V	Hijuelas	32°51'58.25	71°04'33.83	*Quillaja saponaria*	394	Diaspididae	-	MH456783	MH455911
Encarsia sp.	25094	03/12/2015	V	Hijuelas	32°51'58.25	71°04'33.83	*Quillaja saponaria*	394	Diaspididae	-	MH456784	MH455912
Encyrtidae sp.	23829	21/05/2015	IV	La Serena	29°56'22.33	71°08'49.74	*Olea europaea*	120	Coccidae	*Saissetia coffeae*	-	MH455639
Encyrtidae sp.	23955	17/04/2015	V	Quillota	32°50'54.81	71°13'57.89	*Citrus aurantifolia*	158	Diaspididae	*Lepidosaphes beckii*	-	MH455637
Encyrtidae sp.	23956	17/04/2015	V	Quillota	32°50'54.81	71°13'57.89	*Citrus aurantifolia*	158	Diaspididae	*Lepidosaphes beckii*	-	MH455638
Encyrtidae sp.	24053	08/04/2015	V	La Cruz	32°51'20.88	71°11'16.79	*Olea europaea*	195	Diaspididae	*Aspidiotus nerii*	MH456773	MH455640

Species name	Molecular Code	Sample Date	Region	Municipality	Latitud	Longitud	Host plant	Altitude	Family Host	Species Host	GenBank Access COI	GenBank Access 28S
Eupilminae sp.	23787	16/04/2015	V	Hijuelas	32°51'55.35	71°04'01.65	*Schinus latifolius*	471	Coccidae	*Saissetia oleae*	-	MH455926
Eupilminae sp.	23996	01/06/2015	IV	Ovalle	30°32'16.49	71°08'40.31	*Annona cherimola*	286	Coccidae	*Parasaissetia nigra*	-	MH455927
Eupilminae sp.	24080	23/03/2015	Met	Santiago	33°26'27.62	70°38'38.34	*Olea europaea*	595	Coccidae	*Saissetia oleae*	-	MH455928
Eupilminae sp.	24100	27/02/2015	V	Hijuelas	32°51'27.1	71°03'59.58	*Persea americana*	351	Coccidae	*Saissetia oleae*	-	MH455930
Eupilminae sp.	24101	27/02/2015	V	Hijuelas	32°51'27.1	71°03'59.58	*Persea americana*	351	Coccidae	*Saissetia oleae*	-	MH455931
Eupilminae sp.	24933	05/01/2016	VII	Parral	36°08'49.7	71°49'09.76	*Citrus limon*	178	Coccidae	*Saissetia oleae*	-	MH455932
Eupilminae sp.	24949	06/01/2016	VI	San Fernando	34°30'26.87	70°55'19.97	*Schinus latifolius*	313	Coccidae	*Saissetia oleae*	-	MH455933
Eupilminae sp.	24979	05/01/2016	VI	Santa Cruz	34°39'11.4	71°20'37.38	*Olea europaea*	177	Coccidae	*Saissetia oleae*	-	MH455934
Eupilminae sp.	25019	16/02/2016	IV	La Serena	29°55'08.61	71°14'33.31	*Citrus sinensis*	92	Coccidae	*Saissetia oleae*	-	MH455935
Eupilminae sp.	25020	05/01/2016	VIII	San Carlos	36°26'13.64	71°57'45.56	*Olea europaea*	179	Coccidae	*Saissetia oleae*	-	MH455936
Eupilminae sp.	27824	05/01/2016	VIII	San Carlos	36°25'58.85	71°57'42.7	*Maytenus boaria*	181	Coccidae	*Saissetia oleae*	-	MH455937
Eupilminae sp.	27862	05/01/2016	Met	Paine	33°51'17.41	70°45'49.47	*Citrus limon*	375	Coccidae	*Saissetia oleae*	-	MH455938
Hymenoptera	23694	28/05/2015	VII	Linares	35°50'32.15	71°37'00.87	*Quillaja saponaria*	156	Coccidae	*Saissetia oleae*	-	MH456321
Hymenoptera	23733	16/04/2015	V	Hijuelas	32°51'24.77	71°03'51.8	*Persea americana*	370	Coccidae	*Saissetia oleae*	MH456449	MH456322
Hymenoptera	23734	16/04/2015	V	Hijuelas	32°51'24.77	71°03'51.8	*Persea americana*	370	Coccidae	*Saissetia oleae*	MH456450	MH456323
Hymenoptera	23704	28/05/2015	VIII	San Carlos	36°25'58.64	71°57'42.88	*Maytenus boaria*	181	Coccidae	*Saissetia oleae*	MH456448	-
Hymenoptera	23778	22/04/2015	V	Quillota	32°52'19.48	71°11'38.11	*Schinus latifolius*	176	Coccidae	*Saissetia oleae*	MH456451	MH456324
Hymenoptera	23921	01/04/2015	V	Cabildo	32°26'22.3	71°05'49.72	*Persea americana*	173	Coccidae	*Saissetia oleae*	MH456452	MH456325
Hymenoptera	25122	16/12/2015	V	Quillota	32°56'06.20	71°15'28	*Citrus limon*	230	Diaspididae	-	MH456611	-
Hymenoptera	24015	01/04/2015	V	Cabildo	32°26'18.05	71°06'12.46	*Persea americana*	240	Diaspididae	*Saissetia oleae*	MH456582	MH455626
Hymenoptera	24022	26/03/2015	VI	San Fernando	34°30'26.88	70°55'20.31	*Schinus latifolius*	313	Coccidae	*Saissetia oleae*	MH456454	MH456326
Hymenoptera	24835	15/09/2015	V	Hijuelas	32°51'26.27	71°03'54.65	*Persea americana*	368	Coccidae	*Saissetia oleae*	MH456455	MH456332
Hymenoptera	24848	12/10/2015	VI	Coya	34°12'23.5	70°31'50.83	*Citrus sinensis*	786	Coccidae	*Saissetia oleae*	MH456456	MH456333
Hymenoptera	24860	09/09/2015	V	Quillota	32°51'45.95	71°11'23.69	*Persea americana*	172	Diaspididae	*Hemiberlesia lataniae*	MH456583	MH455629
Hymenoptera	24941	16/02/2016	IV	La Serena	29°55'08.61	71°14'33.31	*Hedera helix*	92	Coccidae	*Protopulvinaria pyriformis*	MH456774	MH456315
Hymenoptera	24942	16/02/2016	IV	La Serena	29°55'08.61	71°14'33.31	*Hedera helix*	92	Coccidae	*Protopulvinaria pyriformis*	MH456775	MH456316
Hymenoptera	24960	05/01/2016	VI	Santa Cruz	34°39'11.4	71°20'37.38	*Olea europaea*	177	Coccidae	*Saissetia oleae*	MH456457	MH456336
Hymenoptera	24961	05/01/2016	VI	Santa Cruz	34°39'11.4	71°20'37.38	*Olea europaea*	177	Coccidae	*Saissetia oleae*	MH456458	MH456337
Hymenoptera	24974	05/01/2016	Met	Paine	33°51'05.26	70°45'50.46	*Olea europaea*	380	Coccidae	*Saissetia oleae*	MH456459	MH456340
Hymenoptera	24976	05/01/2016	Met	Paine	33°51'17.41	70°45'49.47	*Citrus limon*	375	Coccidae	*Saissetia oleae*	MH456460	MH456342
Hymenoptera	25029	26/11/2015	XV	Arica	18°34'57.31	69°57'09.14	*Olea europaea*	834	Diaspididae	*Hemiberlesia palmae*	MH456584	MH455630
Hymenoptera	25030	05/01/2016	Met	Paine	33°51'19.8	70°45'21.38	*Hedera helix*	378	Diaspididae	*Aonidiella ensifera*	MH456585	MH455631
Hymenoptera	25047	03/12/2015	V	Hijuelas	32°51'59.46	71°04'33.13	*Cryptocarya alba*	410	Coccidae	*Saissetia oleae*	MH456461	MH456347
Hymenoptera	25048	03/12/2015	V	Hijuelas	32°51'59.46	71°04'33.13	*Cryptocarya alba*	410	Coccidae	*Saissetia oleae*	MH456586	MH455632
Hymenoptera	25069	03/12/2015	V	Hijuelas	32°51'44.15	71°03'55.95	*Persea americana*	459	Coccidae	*Saissetia oleae*	MH456462	MH456348
Hymenoptera	25070	03/12/2015	V	Hijuelas	32°51'44.15	71°03'55.95	*Persea americana*	459	Coccidae	*Saissetia oleae*	MH456463	MH456349
Hymenoptera	25087	03/12/2015	V	Hijuelas	32°51'55.64	71°04'01.6	*Schinus latifolius*	496	Coccidae	*Saissetia oleae*	MH456464	MH456350
Hymenoptera	25088	03/12/2015	V	Hijuelas	32°51'55.64	71°04'01.6	*Schinus latifolius*	496	Coccidae	*Saissetia oleae*	MH456465	MH456351
Hymenoptera	25091	03/12/2015	V	Hijuelas	32°51'27.35	71°04'00.06	*Persea americana*	351	Coccidae	*Saissetia oleae*	MH456466	MH456352
Hymenoptera	25092	03/12/2015	V	Hijuelas	32°51'27.35	71°04'00.06	*Persea americana*	351	Coccidae	*Saissetia oleae*	MH456467	MH456353
Hymenoptera	25097	03/12/2015	V	Hijuelas	32°51'55.64	71°04'01.6	*Schinus latifolius*	496	Coccidae	*Saissetia oleae*	MH456468	MH456354
Hymenoptera	25098	03/12/2015	V	Hijuelas	32°51'55.64	71°04'01.6	*Schinus latifolius*	496	Coccidae	*Saissetia oleae*	MH456469	MH456355
Hymenoptera	25108	05/01/2016	VII	Sagrada Familia	35°07'57.24	71°37'27.61	*Olea europaea*	96	-	-	MH456470	MH456369
Hymenoptera	24088	16/04/2015	V	Hijuelas	32°51'24.77	71°03'51.8	*Persea americana*	370	Coccidae	*Saissetia oleae*	-	MH456327
Hymenoptera	24089	16/04/2015	V	Hijuelas	32°51'24.77	71°03'51.8	*Persea americana*	370	Coccidae	*Saissetia oleae*	-	MH456370
Hymenoptera	25109	07/12/2015	Met	San José de Maipo	33°40'43.15	70°20'27.03	*Maytenus boaria*	1005	Coccidae	*Saissetia oleae*	MH456471	MH456356
Hymenoptera	24099	27/02/2015	V	Hijuelas	32°51'27.1	71°03'59.58	*Persea americana*	351	Coccidae	*Saissetia oleae*	-	MH455627
Hymenoptera	24102	27/02/2015	V	Hijuelas	32°51'27.1	71°03'59.58	*Persea americana*	351	Coccidae	*Saissetia oleae*	-	MH456328
Hymenoptera	24103	27/02/2015	V	Hijuelas	32°51'27.1	71°03'59.58	*Persea americana*	351	Coccidae	*Saissetia oleae*	-	MH456329
Hymenoptera	25128	16/12/2015	V	Quillota	32°56'06.20	71°15'28	*Citrus limon*	230	Coccidae	*Ceroplastes sinensis*	MH456472	MH456358
Hymenoptera	25151	18/12/2015	V	Quillota	32°51'54.4	71°11'10.98	*Schinus latifolius*	185	Coccidae	*Saissetia oleae*	MH456473	MH456359
Hymenoptera	24139	16/04/2015	V	Hijuelas	32°51'55.35	71°04'01.65	*Schinus latifolius*	471	Coccidae	*Saissetia oleae*	-	MH456330
Hymenoptera	24140	16/04/2015	V	Hijuelas	32°51'55.35	71°04'01.65	*Schinus latifolius*	471	Coccidae	*Saissetia oleae*	-	MH456331
Hymenoptera	24148	01/04/2015	V	Cabildo	32°26'18.05	71°06'12.46	*Persea americana*	240	Coccidae	*Saissetia oleae*	-	MH455628

Species name	Molecular Code	Sample Date	Region	Municipality	Latitud	Longitud	Host plant	Altitude	Family Host	Species Host	GenBank Access COI	GenBank Access 28S
Hymenoptera	25152	18/12/2015	V	Quillota	32°51'54.4	71°11'10.98	*Schinus latifolius*	185	Coccidae	*Saissetia oleae*	MH456474	MH456360
Hymenoptera	25155	06/01/2016	V	Putaendo	32°38'26.91	70°43'08.73	*Olea europaea*	790	Diaspididae	*Aspidiotus nerii*	MH456475	MH456361
Hymenoptera	27820	05/01/2016	Met	Paine	33°51'19.8	70°45'21.38	*Hedera helix*	378	Coccidae	*Protopulvinaria pyriformis*	MH456587	MH455633
Hymenoptera	27822	05/01/2016	Met	Paine	33°51'19.8	70°45'21.38	*Hedera helix*	378	Coccidae	*Protopulvinaria pyriformis*	MH456588	MH455634
Hymenoptera	27832	05/01/2016	Met	Paine	33°51'19.8	70°45'21.38	*Hedera helix*	378	Diaspididae	*Aonidiella ensifera*	MH456590	MH455636
Hymenoptera	27838	06/01/2016	V	Catemu	32°47'28.56	70°52'11.09	*Nerium oleander*	499	Coccidae	*Saissetia oleae*	MH456476	MH456362
Hymenoptera	27839	06/01/2016	V	Catemu	32°47'28.56	70°52'11.09	*Nerium oleander*	499	Coccidae	*Saissetia oleae*	MH456477	MH456363
Hymenoptera	27840	06/01/2016	V	Catemu	32°47'28.56	70°52'11.09	*Nerium oleander*	499	Coccidae	*Saissetia oleae*	MH456776	MH456317
Hymenoptera	27841	06/01/2016	V	Catemu	32°47'28.56	70°52'11.09	*Nerium oleander*	499	Coccidae	*Saissetia oleae*	MH456777	MH456318
Hymenoptera	27852	05/01/2016	Met	San Bernardo	33°40'11.39	70°43'19.6	*Nerium oleander*	542	Coccidae	*Saissetia oleae*	MH456478	MH456364
Hymenoptera	27853	05/01/2016	Met	San Bernardo	33°40'11.39	70°43'19.6	*Nerium oleander*	542	Coccidae	*Saissetia oleae*	MH456479	MH456365
Hymenoptera	24913	06/01/2016	VII	Curico	34°58'40.76	71°12'25.47	*Nerium oleander*	234	Coccidae	*Saissetia oleae*	-	MH456334
Hymenoptera	24952	17/02/2016	IV	La Serena	29°56'22.49	71°08'50.13	*Olea europaea*	120	Diaspididae	*Saissetia coffeae*	-	MH456335
Hymenoptera	24972	16/02/2016	IV	Vicuña	30°02'08.3	70°41'48.43	*Vitis vignifera*	632	Coccidae	*Coccus hesperidum*	-	MH456338
Hymenoptera	24973	05/01/2016	Met	Paine	33°51'05.26	70°45'50.46	*Olea europaea*	380	Coccidae	*Saissetia oleae*	-	MH456339
Hymenoptera	24975	05/01/2016	Met	Paine	33°51'17.41	70°45'49.47	*Citrus limon*	375	Coccidae	*Saissetia oleae*	-	MH456341
Hymenoptera	24977	06/01/2016	V	Putaendo	32°37'21.76	70°43'00.69	*Citrus sinensis*	831	Coccidae	*Saissetia oleae*	-	MH456343
Hymenoptera	25007	05/01/2016	Met	Paine	33°51'17.41	70°45'49.47	*Citrus limon*	375	Coccidae	*Saissetia oleae*	-	MH456344
Hymenoptera	25008	05/01/2016	VI	Santa Cruz	34°39'11.4	71°20'37.38	*Olea europaea*	177	Coccidae	*Saissetia oleae*	-	MH456345
Hymenoptera	25046	03/12/2015	V	Hijuelas	32°52'08.53	71°04'29.07	*Persea americana*	470	Coccidae	*Saissetia oleae*	-	MH456346
Hymenoptera	25127	16/12/2015	V	Quillota	32°56'06.20	71°15'28	*Citrus limon*	230	Coccidae	*Ceroplastes sinensis*	-	MH456357
Hymenoptera	25132	16/12/2015	V	Quillota	32°56'06.20	71°15'28	*Citrus limon*	230	Coccidae	*Ceroplastes sinensis*	-	MH456319
Hymenoptera	25133	16/12/2015	V	Quillota	32°56'06.20	71°15'28	*Citrus limon*	230	Coccidae	*Ceroplastes sinensis*	-	MH456320
Hymenoptera	27858	05/01/2016	Met	San Bernardo	33°40'11.39	70°43'19.6	*Nerium oleander*	542	Coccidae	*Saissetia oleae*	-	MH456366
Hymenoptera	27859	05/01/2016	Met	San Bernardo	33°40'11.39	70°43'19.6	*Nerium oleander*	542	Coccidae	*Saissetia oleae*	-	MH456367
Hymenoptera	27863	05/01/2016	Met	Paine	33°51'17.41	70°45'49.47	*Citrus limon*	375	Coccidae	*Saissetia oleae*	-	MH456368
Marietta caridei	23786	22/04/2015	V	Quillota	32°52'19.48	71°11'38.11	*Schinus latifolius*	176	Coccidae	*Saissetia oleae*	-	MH455939
Marietta caridei	24108	27/02/2015	V	Hijuelas	32°51'27.1	71°03'59.58	*Persea americana*	351	Coccidae	*Saissetia oleae*	-	MH455941
Marietta caridei	24109	27/02/2015	V	Hijuelas	32°51'27.1	71°03'59.58	*Persea americana*	351	Coccidae	*Saissetia oleae*	-	MH455942
Marietta caridei	23902	22/04/2015	V	Quillota	32°52'22.21	71°11'40.75	*Citrus sinensis*	165	Coccidae	*Saissetia oleae*	MH456576	MH455940
Marietta caridei	25101	03/12/2015	V	Hijuelas	32°51'43.41	71°03'57.2	*Citrus limon*	425	Coccidae	*Saissetia oleae*	MH456578	MH455946
Marietta caridei	25104	07/12/2015	Met	San José de Maipo	33°40'43.15	70°20'27.03	*Maytenus boaria*	1005	Coccidae	*Saissetia oleae*	MH456577	MH455947
Marietta caridei	24841	09/09/2015	V	Quillota	32°52'19.03	71°11'44.94	*Olea europaea*	166	Coccidae	*Saissetia oleae*	-	MH455943
Marietta caridei	25083	03/12/2015	V	Hijuelas	32°51'44.15	71°03'55.95	*Persea americana*	459	Coccidae	*Saissetia oleae*	-	MH455944
Marietta caridei	25084	03/12/2015	V	Hijuelas	32°51'44.15	71°03'55.95	*Persea americana*	459	Coccidae	*Saissetia oleae*	-	MH455945
Megastigmus transvaalensis	25081	03/12/2015	V	Hijuelas	32°51'55.64	71°04'01.6	*Schinus latifolius*	496	-	-	-	MH455915
Megastigmus transvaalensis	25082	03/12/2015	V	Hijuelas	32°51'55.64	71°04'01.6	*Schinus latifolius*	496	-	-	-	MH455916
Metaphycus anneckei	24097	27/02/2015	V	Hijuelas	32°51'27.1	71°03'59.58	*Persea americana*	351	Coccidae	*Saissetia oleae*	-	MH455949
Metaphycus anneckei	24777	21/09/2015	V	Cabildo	32°28'23.98	71°07'07.61	*Olea europaea*	133	Diaspididae	*Saissetia oleae*	MH456594	MH455950
Metaphycus anneckei	24897	06/01/2016	V	Putaendo	32°37'21.76	70°43'00.69	*Citrus sinensis*	831	Coccidae	*Saissetia oleae*	MH456595	MH455951
Metaphycus anneckei	25006	05/01/2016	Met	Paine	33°51'17.41	70°45'49.47	*Citrus limon*	375	Coccidae	*Saissetia oleae*	MH456596	MH455952
Metaphycus anneckei	25056	03/12/2015	V	Hijuelas	32°51'52.16	71°04'18.45	*Persea americana*	392	Coccidae	-	MH456597	MH455953
Metaphycus anneckei	25123	16/12/2015	V	Quillota	32°56'06.20	71°15'28	*Citrus limon*	230	Coccidae	*Ceroplastes sinensis*	MH456598	MH455954
Metaphycus anneckei	25124	16/12/2015	V	Quillota	32°56'06.20	71°15'28	*Citrus limon*	230	Coccidae	*Ceroplastes sinensis*	MH456599	MH455955
Metaphycus anneckei	25138	18/12/2015	V	Quillota	32°51'54.4	71°11'10.98	*Schinus latifolius*	185	Coccidae	*Saissetia oleae*	MH456600	MH455956
Metaphycus anneckei	27799	06/01/2016	V	Putaendo	32°38'26.91	70°43'08.73	*Olea europaea*	790	Coccidae	*Saissetia oleae*	MH456601	MH455957
Metaphycus anneckei	27800	06/01/2016	V	Putaendo	32°38'26.91	70°43'08.73	*Olea europaea*	790	Coccidae	*Saissetia oleae*	MH456602	MH455958
Metaphycus anneckei	27842	06/01/2016	V	Catemu	32°47'28.56	70°52'11.09	*Nerium oleander*	499	Coccidae	*Saissetia oleae*	MH456603	MH455959
Metaphycus anneckei	27844	06/01/2016	V	Catemu	32°47'28.56	70°52'11.09	*Nerium oleander*	499	Coccidae	*Saissetia oleae*	MH456604	MH455960
Metaphycus anneckei	27846	06/01/2016	V	Catemu	32°47'28.56	70°52'11.09	*Nerium oleander*	499	Coccidae	*Saissetia oleae*	MH456605	MH455961
Metaphycus anneckei	27847	06/01/2016	V	Catemu	32°47'28.56	70°52'11.09	*Nerium oleander*	499	Coccidae	*Saissetia oleae*	MH456606	MH455962
Metaphycus anneckei	27851	06/01/2016	V	Catemu	32°47'28.56	70°52'11.09	*Nerium oleander*	499	Coccidae	*Saissetia oleae*	MH456607	MH455963
Metaphycus anneckei	27860	05/01/2016	Met	San Bernardo	33°40'11.39	70°43'19.6	*Nerium oleander*	542	Coccidae	*Saissetia oleae*	MH456608	MH455964
Metaphycus flavus	23878	06/05/2015	V	Cartagena	33°28'58.44	71°25'10.95	*Schinus latifolius*	218	Coccidae	*Saissetia oleae*	-	MH456195

Species name	Molecular Code	Sample Date	Region	Municipality	Latitud	Longitud	Host plant	Altitude	Family Host	Species Host	GenBank Access COI	GenBank Access 28S
Metaphycus flavus	24143	16/04/2015	V	Hijuelas	32°51'55.35	71°04'01.65	*Schinus latifolius*	471	Coccidae	*Saissetia oleae*	-	MH456198
Metaphycus flavus	24792	10/09/2015	V	Ocoa	32°51'36.49	71°04'47.59	*Persea americana*	329	Coccidae	*Saissetia oleae*	-	MH456201
Metaphycus flavus	24793	09/09/2015	V	Quillota	32°52'19.03	71°11'44.94	*Olea europaea*	166	Coccidae	*Saissetia oleae*	-	MH456202
Metaphycus flavus	24830	21/09/2015	V	Putaendo	32°37'21.80	70°43'00.14	*Citrus clementina*	831	Diaspididae	*Saissetia oleae*	-	MH456205
Metaphycus flavus	24831	21/09/2015	V	Putaendo	32°37'21.80	70°43'00.14	*Citrus clementina*	831	Diaspididae	*Saissetia oleae*	-	MH456206
Metaphycus flavus	23795	22/04/2015	V	Quillota	32°52'19.53	71°11'39.09	*Maytenus boaria*	165	Coccidae	*Saissetia oleae*	MH456723	MH456194
Metaphycus flavus	23887	06/05/2015	V	Casablanca	33°27'25.54	71°23'40.84	*Schinus latifolius*	241	Coccidae	*Saissetia oleae*	MH456724	MH456192
Metaphycus flavus	23940	16/04/2015	V	Hijuelas	32°51'42.44	71°04'17.68	*Persea americana*	367	Coccidae	*Saissetia oleae*	MH456725	MH456196
Metaphycus flavus	23974	21/05/2015	IV	Vicuña	30°02'04.6	70°42'46.14	*Nerium oleander*	616	Coccidae	*Coccus hesperidum*	MH456726	MH456197
Metaphycus flavus	27785	05/01/2016	VIII	San Carlos	36°26'05.37	71°57'44.98	*Hedera helix*	185	Diaspididae	*Aspidiotus nerii*	MH456742	-
Metaphycus flavus	24790	09/09/2015	V	Quillota	32°51'44.98	71°11'23.72	*Persea americana*	172	Coccidae	*Protopulvinaria pyriformis*	MH456727	MH456199
Metaphycus flavus	24791	10/09/2015	V	Ocoa	32°51'36.49	71°04'47.59	*Persea americana*	329	Coccidae	*Saissetia oleae*	MH456728	MH456200
Metaphycus flavus	24820	15/09/2015	V	Hijuelas	32°51'30.92	71°03'48.03	Unknown	392	Coccidae	*Saissetia oleae*	MH456729	MH456203
Metaphycus flavus	24821	15/09/2015	V	Hijuelas	32°51'30.92	71°03'48.03	Unknown	392	Coccidae	*Saissetia oleae*	MH456730	MH456204
Metaphycus flavus	24836	15/09/2015	V	Hijuelas	32°51'30.92	71°03'48.03	Unknown	392	Coccidae	*Saissetia oleae*	MH456731	MH455948
Metaphycus flavus	24837	15/09/2015	V	Hijuelas	32°51'30.92	71°03'48.03	Unknown	392	Coccidae	*Saissetia oleae*	MH456732	MH456207
Metaphycus flavus	24839	10/09/2015	V	Ocoa	32°51'36.49	71°04'47.59	*Persea americana*	329	Coccidae	*Saissetia oleae*	MH456733	MH456208
Metaphycus flavus	24840	09/09/2015	V	Quillota	32°52'19.03	71°11'44.94	*Olea europaea*	166	Coccidae	*Saissetia oleae*	MH456734	MH456209
Metaphycus flavus	24857	15/09/2015	V	Hijuelas	32°51'35.7	71°03'47.75	*Persea americana*	407	Diaspididae	*Aspidiotus nerii*	MH456735	MH456210
Metaphycus flavus	25015	16/02/2016	III	Huasco	28°28'53.84	71°09'28.44	*Olea europaea*	37	Diaspididae	-	MH456778	MH456211
Metaphycus flavus	25033	26/11/2015	XV	Arica	18°34'57.31	69°57'09.14	*Olea europaea*	834	Coccidae	Saissetia coffeae	MH456780	MH456212
Metaphycus flavus	25034	17/11/2015	XIV	Valdivia	39°48'22.55	73°15'03.7	*Choisya ternata*	18	Coccidae	*Pulvinariella mesembryanthemi*	MH456781	MH456213
Metaphycus flavus	25035	17/11/2015	XIV	Valdivia	39°48'22.55	73°15'03.7	*Choisya ternata*	18	Coccidae	*Pulvinariella mesembryanthemi*	MH456782	MH456214
Metaphycus flavus	25036	17/11/2015	XIV	Valdivia	39°48'22.55	73°15'03.7	*Choisya ternata*	18	Coccidae	*Pulvinariella mesembryanthemi*	MH456736	MH456215
Metaphycus flavus	25039	03/12/2015	V	Hijuelas	32°51'59.46	71°04'33.13	*Cryptocarya alba*	410	Coccidae	*Saissetia oleae*	MH456737	MH456216
Metaphycus flavus	25105	03/12/2015	V	Hijuelas	32°51'55.64	71°04'01.6	*Cryptocarya alba*	496	Coccidae	*Protopulvinaria pyriformis*	MH456738	MH456218
Metaphycus flavus	25106	03/12/2015	V	Hijuelas	32°51'55.64	71°04'01.6	*Cryptocarya alba*	496	Coccidae	*Protopulvinaria pyriformis*	MH456739	MH456219
Metaphycus flavus	25117	03/12/2015	V	Hijuelas	32°51'55.64	71°04'01.6	*Cryptocarya alba*	496	Coccidae	*Protopulvinaria pyriformis*	MH456740	MH456220
Metaphycus flavus	25118	03/12/2015	V	Hijuelas	32°51'55.64	71°04'01.6	*Cryptocarya alba*	496	Coccidae	*Protopulvinaria pyriformis*	MH456741	MH456221
Metaphycus flavus	27894	30/01/2015	VI	Chimbarongo	34°38'34.02	70°59'19.22	*Hedera helix*	376	-	-	MH455616	MH456222
Metaphycus flavus	25085	17/11/2015	XIV	Valdivia	39°48'22.55	73°15'03.7	Unknown	18	Diaspididae	*Aonidomytilus sp.*	-	MH456217
Metaphycus helvolus	23728	28/05/2015	VIII	San Carlos	36°25'58.64	71°57'42.88	*Maytenus boaria*	181	Coccidae	*Saissetia oleae*	-	MH455986
Metaphycus helvolus	23729	28/05/2015	VIII	San Carlos	36°25'58.64	71°57'42.88	*Maytenus boaria*	181	Coccidae	*Saissetia oleae*	-	MH455987
Metaphycus helvolus	23730	28/05/2015	VIII	San Carlos	36°25'58.64	71°57'42.88	*Maytenus boaria*	181	Coccidae	*Saissetia oleae*	-	MH455988
Metaphycus helvolus	23731	28/05/2015	VIII	San Carlos	36°25'58.64	71°57'42.88	*Maytenus boaria*	181	Coccidae	*Saissetia oleae*	-	MH455989
Metaphycus helvolus	23732	28/05/2015	VIII	San Carlos	36°25'58.64	71°57'42.88	*Maytenus boaria*	181	Coccidae	*Saissetia oleae*	-	MH455990
Metaphycus helvolus	23744	28/05/2015	VII	Linares	35°50'32.15	71°37'00.87	*Quillaja saponaria*	156	Coccidae	*Saissetia oleae*	-	MH455991
Metaphycus helvolus	23745	28/05/2015	VII	Linares	35°50'32.15	71°37'00.87	*Quillaja saponaria*	156	Coccidae	*Saissetia oleae*	-	MH455992
Metaphycus helvolus	23746	28/05/2015	VII	Linares	35°50'32.15	71°37'00.87	*Quillaja saponaria*	156	Coccidae	*Saissetia oleae*	-	MH455993
Metaphycus helvolus	23760	28/05/2015	VII	Parral	36°08'49.46	71°49'09.33	*Citrus limon*	179	Coccidae	*Saissetia oleae*	-	MH455994
Metaphycus helvolus	23761	02/07/2015	I	Pisagua	19°35'47.72	70°12'41.7	*Acacia retinodes*	12	Diaspididae	-	-	MH455995
Metaphycus helvolus	23773	28/05/2015	VII	Linares	35°50'32.15	71°37'00.87	*Nerium oleander*	156	Coccidae	*Saissetia oleae*	-	MH455996
Metaphycus helvolus	23779	01/04/2015	V	Cabildo	32°26'22.3	71°05'49.72	*Persea americana*	173	Diaspididae	*Saissetia oleae*	-	MH455997
Metaphycus helvolus	23789	22/04/2015	V	Quillota	32°52'19.48	71°11'38.11	*Schinus latifolius*	176	Coccidae	*Saissetia oleae*	-	MH455998
Metaphycus helvolus	23790	22/04/2015	V	Quillota	32°52'19.48	71°11'38.11	*Schinus latifolius*	176	Coccidae	*Saissetia oleae*	-	MH455999
Metaphycus helvolus	23791	22/04/2015	V	Quillota	32°52'19.48	71°11'38.11	*Schinus latifolius*	176	Coccidae	*Saissetia oleae*	-	MH455977
Metaphycus helvolus	23792	22/04/2015	V	Quillota	32°52'19.48	71°11'38.11	*Schinus latifolius*	176	Coccidae	*Saissetia oleae*	-	MH456000
Metaphycus helvolus	23793	26/03/2015	VII	Sagrada Familia	35°02'04.84	71°18'23.49	*Aristotelia chilensis*	200	Diaspididae	*Hemiberlesia rapax*	-	MH455975
Metaphycus helvolus	23796	22/04/2015	V	Quillota	32°52'19.59	71°11'44.67	*Olea europaea*	159	Coccidae	*Saissetia oleae*	-	MH456001
Metaphycus helvolus	23797	22/04/2015	V	Quillota	32°52'19.59	71°11'44.67	*Olea europaea*	159	Coccidae	*Saissetia oleae*	-	MH456002
Metaphycus helvolus	23805	22/04/2015	V	Quillota	32°52'19.59	71°11'44.67	*Olea europaea*	159	Coccidae	*Saissetia oleae*	-	MH456003
Metaphycus helvolus	23806	22/04/2015	V	Quillota	32°52'19.59	71°11'44.67	*Olea europaea*	159	Coccidae	*Saissetia oleae*	-	MH456004
Metaphycus helvolus	23807	22/04/2015	V	Quillota	32°52'19.59	71°11'44.67	*Olea europaea*	159	Coccidae	*Saissetia oleae*	-	MH456005
Metaphycus helvolus	23808	22/04/2015	V	Quillota	32°52'19.59	71°11'44.67	*Olea europaea*	159	Coccidae	*Saissetia oleae*	-	MH456006

Species name	Molecular Code	Sample Date	Region	Municipality	Latitud	Longitud	Host plant	Altitude	Family Host	Species Host	GenBank Access COI	GenBank Access 28S
Metaphycus helvolus	23809	19/03/2015	VI	Pelequen	34°26'05.43	70°55'22.88	*Citrus* sp.	278	Coccidae	*Saissetia oleae*	-	MH455970
Metaphycus helvolus	23810	19/03/2015	VI	Pelequen	34°26'05.43	70°55'22.88	*Citrus* sp.	278	Coccidae	*Saissetia oleae*	-	MH456007
Metaphycus helvolus	23815	19/03/2015	VI	Pelequen	34°26'05.43	70°55'22.88	*Citrus* sp.	278	Coccidae	*Saissetia oleae*	-	MH456008
Metaphycus helvolus	23816	19/03/2015	VI	Pelequen	34°26'05.43	70°55'22.88	*Citrus* sp.	278	Coccidae	*Saissetia oleae*	-	MH456009
Metaphycus helvolus	23818	28/05/2015	VIII	San Carlos	36°25'58.64	71°57'42.88	*Maytenus boaria*	181	Coccidae	*Saissetia oleae*	-	MH456010
Metaphycus helvolus	23819	28/05/2015	VIII	San Carlos	36°25'58.64	71°57'42.88	*Maytenus boaria*	181	Coccidae	*Saissetia oleae*	-	MH456011
Metaphycus helvolus	23834	28/05/2015	VII	Parral	36°08'49.46	71°49'09.33	*Citrus limon*	179	Coccidae	*Saissetia oleae*	-	MH456012
Metaphycus helvolus	23835	28/05/2015	VII	Parral	36°08'49.46	71°49'09.33	*Citrus limon*	179	Coccidae	*Saissetia oleae*	-	MH455972
Metaphycus helvolus	23841	28/05/2015	VII	Parral	36°08'49.46	71°49'09.33	*Citrus limon*	179	Coccidae	*Saissetia oleae*	-	MH456013
Metaphycus helvolus	23842	28/05/2015	VII	Parral	36°08'49.46	71°49'09.33	*Citrus limon*	179	Coccidae	*Saissetia oleae*	-	MH456014
Metaphycus helvolus	23843	14/05/2015	Met	La Pintana	33°34'07.93	70°38'03.37	*Olea europaea*	623	Coccidae	*Saissetia oleae*	-	MH456015
Metaphycus helvolus	23844	14/05/2015	Met	La Pintana	33°34'07.93	70°38'03.37	*Olea europaea*	623	Coccidae	*Saissetia oleae*	-	MH456016
Metaphycus helvolus	23847	28/05/2015	VII	Parral	36°08'49.46	71°49'09.33	*Citrus limon*	179	Coccidae	*Saissetia oleae*	-	MH456017
Metaphycus helvolus	23848	28/05/2015	VII	Parral	36°08'49.46	71°49'09.33	*Citrus limon*	179	Coccidae	*Saissetia oleae*	-	MH456018
Metaphycus helvolus	23849	02/06/2015	IV	Monte Patria	30°43'49.4	70°52'53.19	*Persea americana*	565	Diaspididae	*Hemiberlesia lataniae*	-	MH456019
Metaphycus helvolus	23863	19/03/2015	VI	Pelequen	34°26'05.43	70°55'22.88	Unknown	278	Coccidae	*Saissetia oleae*	-	MH456020
Metaphycus helvolus	23864	19/03/2015	VI	Pelequen	34°26'05.43	70°55'22.88	Unknown	278	Coccidae	*Saissetia oleae*	-	MH456021
Metaphycus helvolus	23867	19/03/2015	VI	Pelequen	34°26'05.43	70°55'22.88	Unknown	278	Coccidae	*Saissetia oleae*	-	MH456022
Metaphycus helvolus	23880	06/05/2015	V	Cartagena	33°28'58.44	71°25'10.95	*Schinus latifolius*	218	Coccidae	*Saissetia oleae*	-	MH455978
Metaphycus helvolus	23888	06/05/2015	V	Cartagena	33°28'58.44	71°25'10.95	*Schinus latifolius*	218	Coccidae	*Saissetia oleae*	-	MH456023
Metaphycus helvolus	23889	06/05/2015	V	Cartagena	33°28'58.44	71°25'10.95	*Schinus latifolius*	218	Coccidae	*Saissetia oleae*	-	MH456024
Metaphycus helvolus	23890	06/05/2015	V	Cartagena	33°28'58.44	71°25'10.95	*Schinus latifolius*	218	Coccidae	*Saissetia oleae*	-	MH456025
Metaphycus helvolus	23891	06/05/2015	V	Cartagena	33°28'58.44	71°25'10.95	*Schinus latifolius*	218	Coccidae	*Saissetia oleae*	-	MH456026
Metaphycus helvolus	23899	22/04/2015	V	Quillota	32°52'22.21	71°11'40.75	*Citrus sinensis*	165	Coccidae	*Saissetia oleae*	-	MH456027
Metaphycus helvolus	23900	22/04/2015	V	Quillota	32°52'22.21	71°11'40.75	*Citrus sinensis*	165	Coccidae	*Saissetia oleae*	-	MH456028
Metaphycus helvolus	23911	15/04/2015	V	Quillota	32°53'46.38	71°12'33.68	*Hedera helix*	140	Coccidae	*Protopulvinaria pyriformis*	-	MH456029
Metaphycus helvolus	23966	22/04/2015	V	Quillota	32°52'24.58	71°11'45.05	*Citrus sinensis*	158	Coccidae	*Saissetia oleae*	-	MH456030
Metaphycus helvolus	23969	26/05/2015	IV	Coquimbo	29°57'20.09	71°20'10.99	*Olea europaea*	6	Coccidae	*Saissetia coffeae*	-	MH456031
Metaphycus helvolus	23971	28/05/2015	VII	Curico	34°58'40.96	71°12'24.27	*Nerium oleander*	234	Coccidae	*Saissetia oleae*	-	MH455985
Metaphycus helvolus	23975	28/05/2015	VII	Linares	35°50'32.15	71°37'00.87	*Quillaja saponaria*	156	Coccidae	*Saissetia oleae*	-	MH456032
Metaphycus helvolus	23976	28/05/2015	VII	Linares	35°50'32.15	71°37'00.87	*Quillaja saponaria*	156	Coccidae	*Saissetia oleae*	-	MH455984
Metaphycus helvolus	23982	28/05/2015	VII	Linares	35°50'32.15	71°37'00.87	*Quillaja saponaria*	156	Coccidae	*Saissetia oleae*	-	MH456033
Metaphycus helvolus	23987	28/05/2015	VII	Parral	36°08'57.53	71°49'16.5	*Nerium oleander*	182	Coccidae	*Saissetia oleae*	-	MH456034
Metaphycus helvolus	24000	01/06/2015	IV	Ovalle	30°32'16.49	71°08'40.31	*Annona cherimola*	286	Coccidae	*Parasaissetia nigra*	-	MH456035
Metaphycus helvolus	24003	01/06/2015	IV	Ovalle	30°32'16.49	71°08'40.31	*Annona cherimola*	286	Coccidae	*Parasaissetia nigra*	-	MH456036
Metaphycus helvolus	24004	01/06/2015	IV	Ovalle	30°32'16.49	71°08'40.31	*Annona cherimola*	286	Coccidae	*Parasaissetia nigra*	-	MH456037
Metaphycus helvolus	24033	19/03/2015	VI	Pelequen	34°26'05.43	70°55'22.88	Unknown	278	Coccidae	*Saissetia oleae*	-	MH456038
Metaphycus helvolus	24034	19/03/2015	VI	Pelequen	34°26'05.43	70°55'22.88	Unknown	278	Coccidae	*Saissetia oleae*	-	MH456039
Metaphycus helvolus	24086	16/04/2015	V	Hijuelas	32°51'24.77	71°03'51.8	*Persea americana*	370	Coccidae	*Saissetia oleae*	-	MH456040
Metaphycus helvolus	24094	16/04/2015	V	Hijuelas	32°51'24.77	71°03'51.8	*Persea americana*	370	Coccidae	*Saissetia oleae*	-	MH456041
Metaphycus helvolus	24095	16/04/2015	V	Hijuelas	32°51'24.77	71°03'51.8	*Persea americana*	370	Coccidae	*Saissetia oleae*	-	MH456042
Metaphycus helvolus	24144	16/04/2015	V	Hijuelas	32°51'55.35	71°04'01.65	*Schinus latifolius*	471	Coccidae	*Saissetia oleae*	-	MH456043
Metaphycus helvolus	24773	12/10/2015	VI	Rancagua	34°10'01.28	70°44'01.55	*Hedera helix*	508	Coccidae	*Protopulvinaria pyriformis*	-	MH456044
Metaphycus helvolus	24780	20/09/2015	Met	Til Til	33°06'50.88	70°55'39.27	*Nerium oleander*	564	Coccidae	*Saissetia oleae*	-	MH456045
Metaphycus helvolus	24781	20/09/2015	Met	Til Til	33°06'50.88	70°55'39.27	*Nerium oleander*	564	Coccidae	*Saissetia oleae*	-	MH456046
Metaphycus helvolus	24797	12/10/2015	VI	Rancagua	34°10'01.28	70°44'01.55	*Hedera helix*	508	Coccidae	*Protopulvinaria pyriformis*	-	MH456047
Metaphycus helvolus	24798	12/10/2015	VI	Rancagua	34°10'01.28	70°44'01.55	*Hedera helix*	508	Coccidae	*Protopulvinaria pyriformis*	-	MH456048
Metaphycus helvolus	24810	09/10/2015	VI	Rengo	34°21'22.75	70°50'45.48	*Nerium oleander*	338	Coccidae	*Saissetia oleae*	-	MH455983
Metaphycus helvolus	24811	09/10/2015	VI	Rengo	34°21'22.75	70°50'45.48	*Nerium oleander*	338	Coccidae	*Saissetia oleae*	-	MH456049
Metaphycus helvolus	24812	21/09/2015	V	Cabildo	32°28'23.98	71°07'07.61	*Olea europaea*	133	Diaspididae	*Saissetia oleae*	-	MH456050
Metaphycus helvolus	24813	21/09/2015	V	Cabildo	32°28'23.98	71°07'07.61	*Olea europaea*	133	Diaspididae	*Saissetia oleae*	-	MH456051
Metaphycus helvolus	24814	21/09/2015	V	Putaendo	32°37'21.80	70°43'00.14	*Citrus clementina*	831	Diaspididae	*Saissetia oleae*	-	MH455980
Metaphycus helvolus	24815	21/09/2015	V	Putaendo	32°37'21.80	70°43'00.14	*Citrus clementina*	831	Diaspididae	*Saissetia oleae*	-	MH456052
Metaphycus helvolus	24818	15/09/2015	V	Hijuelas	32°51'26.27	71°03'54.65	*Persea americana*	368	Coccidae	*Saissetia oleae*	-	MH456053

Species name	Molecular Code	Sample Date	Region	Municipality	Latitud	Longitud	Host plant	Altitude	Family Host	Species Host	GenBank Access COI	GenBank Access 28S
Metaphycus helvolus	24819	15/09/2015	V	Hijuelas	32°51'26.27	71°03'54.65	*Persea americana*	368	Coccidae	*Saissetia oleae*	-	MH456054
Metaphycus helvolus	24822	10/09/2015	V	Ocoa	32°51'36.49	71°04'47.59	*Persea americana*	329	Coccidae	*Saissetia oleae*	-	MH455981
Metaphycus helvolus	24823	10/09/2015	V	Ocoa	32°51'36.49	71°04'47.59	*Persea americana*	329	Coccidae	*Saissetia oleae*	-	MH455974
Metaphycus helvolus	24824	09/09/2015	V	Quillota	32°52'19.03	71°11'44.94	*Olea europaea*	166	Coccidae	*Saissetia oleae*	-	MH456055
Metaphycus helvolus	24825	09/09/2015	V	Quillota	32°52'19.03	71°11'44.94	*Olea europaea*	166	Coccidae	*Saissetia oleae*	-	MH456056
Metaphycus helvolus	24828	09/10/2015	VI	Rengo	34°21'22.75	70°50'45.48	*Nerium oleander*	338	Coccidae	*Saissetia oleae*	-	MH456057
Metaphycus helvolus	24832	20/09/2015	Met	Til Til	33°06'50.88	70°55'39.27	*Nerium oleander*	564	Coccidae	*Saissetia oleae*	-	MH456058
Metaphycus helvolus	24844	09/09/2015	V	Quillota	32°52'31.11	71°11'31.91	*Hedera helix*	167	Coccidae	*Protopulvinaria pyriformis*	-	MH456059
Metaphycus helvolus	24854	21/09/2015	V	Putaendo	32°37'21.80	70°43'00.14	*Citrus clementina*	831	Diaspididae	*Saissetia oleae*	-	MH456060
Metaphycus helvolus	24868	15/09/2015	V	Hijuelas	32°51'30.92	71°03'48.03	Unknown	392	Coccidae	*Saissetia oleae*	-	MH456061
Metaphycus helvolus	24869	15/09/2015	V	Hijuelas	32°51'30.92	71°03'48.03	Unknown	392	Coccidae	*Saissetia oleae*	-	MH456062
Metaphycus helvolus	24870	15/09/2015	V	Hijuelas	32°51'26.27	71°03'54.65	*Persea americana*	368	Coccidae	*Saissetia oleae*	-	MH456063
Metaphycus helvolus	24871	15/09/2015	V	Hijuelas	32°51'26.27	71°03'54.65	*Persea americana*	368	Coccidae	*Saissetia oleae*	-	MH456064
Metaphycus helvolus	24872	21/09/2015	V	Cabildo	32°26'30.40	71°06'03.05	*Persea americana*	181	Coccidae	*Saissetia oleae*	-	MH456065
Metaphycus helvolus	24873	21/09/2015	V	Cabildo	32°26'30.40	71°06'03.05	*Persea americana*	181	Coccidae	*Saissetia oleae*	-	MH456066
Metaphycus helvolus	24876	07/10/2015	VII	Parral	36°08'57.53	71°49'16.5	*Nerium oleander*	182	Coccidae	*Saissetia oleae*	-	MH456067
Metaphycus helvolus	24877	07/10/2015	VII	Parral	36°08'57.53	71°49'16.5	*Nerium oleander*	182	Coccidae	*Saissetia oleae*	-	MH456068
Metaphycus helvolus	24883	09/09/2015	V	Quillota	32°52'19.03	71°11'44.94	*Olea europaea*	166	Coccidae	*Saissetia oleae*	-	MH456069
Metaphycus helvolus	24884	09/09/2015	V	Quillota	32°52'19.03	71°11'44.94	*Olea europaea*	166	Coccidae	*Saissetia oleae*	-	MH456070
Metaphycus helvolus	24966	17/02/2016	IV	La Serena	29°56'22.49	71°08'50.13	*Olea europaea*	120	Diaspididae	*Saissetia coffeae*	-	MH456071
Metaphycus helvolus	24993	06/01/2016	VI	San Fernando	34°30'26.87	70°55'19.97	*Schinus latifolius*	313	Coccidae	*Saissetia oleae*	-	MH456072
Metaphycus helvolus	24997	17/02/2016	IV	La Serena	29°56'22.49	71°08'50.13	*Olea europaea*	120	Diaspididae	*Saissetia coffeae*	-	MH456073
Metaphycus helvolus	25102	03/12/2015	V	Hijuelas	32°51'55.64	71°04'01.6	*Cryptocarya alba*	496	Coccidae	*Protopulvinaria pyriformis*	-	MH456074
Metaphycus helvolus	25103	03/12/2015	V	Hijuelas	32°51'55.64	71°04'01.6	*Cryptocarya alba*	496	Coccidae	*Protopulvinaria pyriformis*	-	MH456075
Metaphycus helvolus	25111	07/12/2015	Met	San José de Maipo	33°40'43.15	70°20'27.03	*Maytenus boaria*	1005	Coccidae	*Saissetia oleae*	-	MH456076
Metaphycus helvolus	25112	07/12/2015	Met	San José de Maipo	33°40'43.15	70°20'27.03	*Maytenus boaria*	1005	Coccidae	*Saissetia oleae*	-	MH456077
Metaphycus helvolus	27904	03/09/2015	V	-	-	-	*Persea americana*	-	-	-	-	MH456078
Metaphycus lounsburyi	23785	22/04/2015	V	Quillota	32°52'19.48	71°11'38.11	*Schinus latifolius*	176	Coccidae	*Saissetia oleae*	-	MH456128
Metaphycus lounsburyi	23830	21/05/2015	IV	La Serena	29°56'22.33	71°08'49.74	*Olea europaea*	120	Coccidae	*Saissetia coffeae*	-	MH456129
Metaphycus lounsburyi	23901	22/04/2015	V	Quillota	32°52'22.21	71°11'40.75	*Citrus sinensis*	165	Coccidae	*Saissetia oleae*	-	MH456132
Metaphycus lounsburyi	24082	23/03/2015	Met	Santiago	33°26'27.62	70°38'38.34	*Olea europaea*	595	Coccidae	*Saissetia oleae*	-	MH456139
Metaphycus lounsburyi	24083	23/03/2015	Met	Santiago	33°26'27.62	70°38'38.34	*Olea europaea*	595	Coccidae	*Saissetia oleae*	-	MH456140
Metaphycus lounsburyi	24096	27/02/2015	V	Hijuelas	32°51'27.1	71°03'59.58	*Persea americana*	351	Coccidae	*Saissetia oleae*	-	MH456141
Metaphycus lounsburyi	24145	26/03/2015	VI	Placilla	34°37'30.91	71°07'27.14	*Citrus sinensis*	254	Coccidae	*Saissetia oleae*	-	MH456142
Metaphycus lounsburyi	24892	05/01/2016	VIII	San Carlos	36°26'13.64	71°57'45.56	*Olea europaea*	179	Coccidae	*Saissetia oleae*	-	MH456143
Metaphycus lounsburyi	24893	05/01/2016	VIII	San Carlos	36°26'13.64	71°57'45.56	*Olea europaea*	179	Coccidae	*Saissetia oleae*	-	MH456144
Metaphycus lounsburyi	24894	05/01/2016	VI	Santa Cruz	34°38'20.8	71°21'58.39	*Citrus sinensis*	174	Coccidae	*Saissetia oleae*	-	MH456145
Metaphycus lounsburyi	24895	05/01/2016	VI	Santa Cruz	34°38'20.8	71°21'58.39	*Citrus sinensis*	174	Coccidae	*Saissetia oleae*	-	MH456146
Metaphycus lounsburyi	24905	05/01/2016	VIII	San Carlos	36°26'13.64	71°57'45.56	*Olea europaea*	179	Coccidae	*Saissetia oleae*	-	MH456147
Metaphycus lounsburyi	23717	28/05/2015	VII	Parral	36°08'57.53	71°49'16.5	*Nerium oleander*	182	Coccidae	*Saissetia oleae*	MH456508	-
Metaphycus lounsburyi	24931	06/01/2016	V	Putaendo	32°37'21.76	70°43'00.69	*Citrus sinensis*	831	Coccidae	-	MH456519	-
Metaphycus lounsburyi	23836	14/05/2015	Met	La Pintana	33°34'07.93	70°38'03.37	*Olea europaea*	623	Coccidae	*Saissetia oleae*	MH456509	MH456130
Metaphycus lounsburyi	25144	25/11/2015	X	Frutillar	41°08'08.03	73°01'37.14	*Ilex* sp.	58	Coccidae	*Aonidomytilus sp.*	MH456536	-
Metaphycus lounsburyi	23861	19/03/2015	VI	Pelequen	34°26'05.43	70°55'22.88	Unknown	278	Coccidae	*Saissetia oleae*	MH456510	MH456131
Metaphycus lounsburyi	23862	19/03/2015	VI	Pelequen	34°26'05.43	70°55'22.88	Unknown	278	Coccidae	*Saissetia oleae*	MH456511	MH456191
Metaphycus lounsburyi	23925	01/04/2015	V	Cabildo	32°26'22.3	71°05'49.72	*Persea americana*	173	Coccidae	*Saissetia oleae*	MH456512	MH456133
Metaphycus lounsburyi	23926	01/04/2015	V	Cabildo	32°26'22.3	71°05'49.72	*Persea americana*	173	Coccidae	*Saissetia oleae*	MH456513	MH456134
Metaphycus lounsburyi	23965	22/04/2015	V	Quillota	32°52'24.58	71°11'45.05	*Citrus sinensis*	158	Coccidae	*Saissetia oleae*	MH456514	MH456135
Metaphycus lounsburyi	24944	05/01/2016	VII	Parral	36°08'49.7	71°49'09.76	*Citrus limon*	178	Coccidae	*Saissetia oleae*	-	MH456150
Metaphycus lounsburyi	24945	05/01/2016	VII	Parral	36°08'49.7	71°49'09.76	*Citrus limon*	178	Coccidae	*Saissetia oleae*	-	MH456151
Metaphycus lounsburyi	24018	08/04/2015	V	La Cruz	32°51'29.52	71°11'19.04	*Persea americana*	186	Coccidae	*Protopulvinaria pyriformis*	MH456515	MH456136
Metaphycus lounsburyi	24019	08/04/2015	V	La Cruz	32°51'29.52	71°11'19.04	*Persea americana*	186	Coccidae	*Protopulvinaria pyriformis*	MH456516	MH456137
Metaphycus lounsburyi	24020	01/04/2015	V	Cabildo	32°26'18.05	71°06'12.46	*Persea americana*	240	Diaspididae	*Saissetia oleae*	MH456517	MH456138
Metaphycus lounsburyi	24951	05/01/2016	VI	Santa Cruz	34°39'11.4	71°20'37.38	*Olea europaea*	177	Coccidae	*Saissetia oleae*	-	MH456127

Species name	Molecular Code	Sample Date	Region	Municipality	Latitud	Longitud	Host plant	Altitude	Family Host	Species Host	GenBank Access COI	GenBank Access 28S
Metaphycus lounsburyi	24906	05/01/2016	VIII	San Carlos	36°26'13.64	71°57'45.56	*Olea europaea*	179	Coccidae	*Saissetia oleae*	MH456518	MH456148
Metaphycus lounsburyi	24932	06/01/2016	V	Putaendo	32°37'21.76	70°43'00.69	*Citrus sinensis*	831	Coccidae	*Saissetia oleae*	MH456520	MH456149
Metaphycus lounsburyi	24946	05/01/2016	VI	Santa Cruz	34°39'11.4	71°20'37.38	*Olea europaea*	177	Coccidae	*Saissetia oleae*	MH456521	MH456152
Metaphycus lounsburyi	24947	05/01/2016	VI	Santa Cruz	34°39'11.4	71°20'37.38	*Olea europaea*	177	Coccidae	*Saissetia oleae*	MH456522	MH456153
Metaphycus lounsburyi	24950	05/01/2016	VI	Santa Cruz	34°39'11.4	71°20'37.38	*Olea europaea*	177	Coccidae	*Saissetia oleae*	MH456523	MH456154
Metaphycus lounsburyi	24962	06/01/2016	V	Putaendo	32°37'21.76	70°43'00.69	*Citrus sinensis*	831	Coccidae	*Saissetia oleae*	-	MH456155
Metaphycus lounsburyi	24963	06/01/2016	V	Putaendo	32°37'21.76	70°43'00.69	*Citrus sinensis*	831	Coccidae	*Saissetia oleae*	MH456524	MH456156
Metaphycus lounsburyi	24988	05/01/2016	Met	Paine	33°51'05.26	70°45'50.46	*Olea europaea*	380	Coccidae	*Saissetia oleae*	MH456525	MH456158
Metaphycus lounsburyi	25038	03/12/2015	V	Hijuelas	32°51'59.46	71°04'33.13	*Cryptocarya alba*	410	Coccidae	*Saissetia oleae*	MH456527	MH456164
Metaphycus lounsburyi	25043	03/12/2015	V	Hijuelas	32°52'08.53	71°04'29.07	*Persea americana*	470	Coccidae	*Saissetia oleae*	MH456528	MH456165
Metaphycus lounsburyi	25057	03/12/2015	V	Hijuelas	32°51'55.64	71°04'01.6	*Schinus latifolius*	496	Coccidae	*Saissetia oleae*	MH456529	MH456166
Metaphycus lounsburyi	24987	16/02/2016	IV	Vicuña	30°02'08.3	70°41'48.43	*Vitis vignifera*	632	Coccidae	*Coccus hesperidum*	-	MH456157
Metaphycus lounsburyi	25061	03/12/2015	V	Hijuelas	32°51'27.35	71°04'00.06	*Persea americana*	351	Coccidae	*Saissetia oleae*	MH456530	MH456169
Metaphycus lounsburyi	24989	05/01/2016	Met	Paine	33°51'05.26	70°45'50.46	*Olea europaea*	380	Coccidae	*Saissetia oleae*	-	MH456159
Metaphycus lounsburyi	24990	05/01/2016	Met	Paine	33°51'17.41	70°45'49.47	*Citrus limon*	375	Coccidae	*Saissetia oleae*	-	MH456160
Metaphycus lounsburyi	25062	03/12/2015	V	Hijuelas	32°51'27.35	71°04'00.06	*Persea americana*	351	Coccidae	*Saissetia oleae*	MH456531	MH456170
Metaphycus lounsburyi	25065	03/12/2015	V	Hijuelas	32°51'44.15	71°03'55.95	*Persea americana*	459	Coccidae	*Saissetia oleae*	MH456532	MH456172
Metaphycus lounsburyi	25099	03/12/2015	V	Hijuelas	32°51'44.15	71°03'55.95	*Persea americana*	459	Coccidae	*Saissetia oleae*	MH456533	MH456173
Metaphycus lounsburyi	25100	03/12/2015	V	Hijuelas	32°51'44.15	71°03'55.95	*Persea americana*	459	Coccidae	*Saissetia oleae*	MH456534	MH456174
Metaphycus lounsburyi	25143	25/11/2015	X	Frutillar	41°08'08.03	73°01'37.14	*Ilex* sp.	58	Coccidae	*Aonidomytilus* sp.	MH456535	MH456175
Metaphycus lounsburyi	27791	05/01/2016	VIII	San Carlos	36°26'05.37	71°57'44.98	*Hedera helix*	185	Diaspididae	*Aspidiotus nerii*	MH456537	MH456176
Metaphycus lounsburyi	27804	05/01/2016	VII	Linares	35°50'32.02	71°37'01.25	*Nerium oleander*	156	Coccidae	*Saissetia oleae*	MH456538	MH456180
Metaphycus lounsburyi	25010	06/01/2016	VI	San Fernando	34°30'26.87	70°55'19.97	*Schinus latifolius*	313	Coccidae	*Saissetia oleae*	-	MH456162
Metaphycus lounsburyi	27805	07/01/2016	VII	Curico	34°59'01.09	71°13'17.73	*Citrus sinensis*	223	Coccidae	*Saissetia oleae*	MH456539	MH456181
Metaphycus lounsburyi	25037	17/11/2015	XIV	Valdivia	39°48'22.55	73°15'03.7	*Choisya ternata*	18	Coccidae	*Pulvinariella mesembryanthemi*	-	MH456163
Metaphycus lounsburyi	25058	03/12/2015	V	Hijuelas	32°51'55.64	71°04'01.6	*Schinus latifolius*	496	Coccidae	*Saissetia oleae*	-	MH456167
Metaphycus lounsburyi	25059	03/12/2015	V	Hijuelas	32°51'52.16	71°04'18.45	*Persea americana*	392	Coccidae	-	-	MH456168
Metaphycus lounsburyi	25064	03/12/2015	V	Hijuelas	32°51'44.15	71°03'55.95	*Persea americana*	459	Coccidae	*Saissetia oleae*	-	MH456171
Metaphycus lounsburyi	27801	06/01/2016	V	Putaendo	32°38'26.91	70°43'08.73	*Olea europaea*	790	Coccidae	*Saissetia oleae*	-	MH456177
Metaphycus lounsburyi	27802	06/01/2016	V	Putaendo	32°38'26.91	70°43'08.73	*Olea europaea*	790	Coccidae	*Saissetia oleae*	-	MH456178
Metaphycus lounsburyi	27803	05/01/2016	VII	Linares	35°50'32.02	71°37'01.25	*Nerium oleander*	156	Coccidae	*Saissetia oleae*	-	MH456179
Metaphycus lounsburyi	27821	05/01/2016	Met	Paine	33°51'19.8	70°45'21.38	*Hedera helix*	378	Coccidae	*Protopulvinaria pyriformis*	-	MH456182
Metaphycus lounsburyi	27845	06/01/2016	V	Catemu	32°47'28.56	70°52'11.09	*Nerium oleander*	499	Coccidae	*Saissetia oleae*	-	MH456183
Metaphycus lounsburyi	27848	06/01/2016	V	Catemu	32°47'28.56	70°52'11.09	*Nerium oleander*	499	Coccidae	*Saissetia oleae*	-	MH456184
Metaphycus lounsburyi	27849	06/01/2016	V	Catemu	32°47'28.56	70°52'11.09	*Nerium oleander*	499	Coccidae	*Saissetia oleae*	-	MH456185
Metaphycus lounsburyi	27850	06/01/2016	V	Catemu	32°47'28.56	70°52'11.09	*Nerium oleander*	499	Coccidae	*Saissetia oleae*	-	MH456186
Metaphycus lounsburyi	27867	05/01/2016	Met	Paine	33°51'17.41	70°45'49.47	*Citrus limon*	375	Coccidae	*Saissetia oleae*	-	MH456187
Metaphycus lounsburyi	27868	05/01/2016	Met	Paine	33°51'17.41	70°45'49.47	*Citrus limon*	375	Coccidae	*Saissetia oleae*	-	MH456188
Metaphycus lounsburyi	27869	05/01/2016	Met	Paine	33°51'17.41	70°45'49.47	*Citrus limon*	375	Coccidae	*Saissetia oleae*	-	MH456189
Metaphycus lounsburyi	27870	05/01/2016	Met	Paine	33°51'17.41	70°45'49.47	*Citrus limon*	375	Coccidae	*Saissetia oleae*	-	MH456190
Metaphycus sp.	24783	13/09/2015	V	Alborrogo	33°21'56.3	71°40'14.22	*Tristerix tetrandrus*	40	Coccidae	*Saissetia oleae*	-	MH455969
Metaphycus sp.	24054	22/04/2015	V	Quillota	32°52'28.38	71°11'39.94	*Persea americana*	163	Diaspididae	*Hemiberlesia lataniae*	MH456593	MH455965
Metaphycus sp.	24055	22/04/2015	V	Quillota	32°52'28.38	71°11'39.94	*Persea americana*	163	Diaspididae	*Hemiberlesia lataniae*	MH456591	MH455966
Metaphycus sp.	24898	06/01/2016	V	Putaendo	32°37'21.76	70°43'00.69	*Citrus sinensis*	831	Coccidae	*Saissetia oleae*	MH456592	MH455967
Metaphycus sp.	25042	25/11/2015	X	Frutillar	41°08'08.03	73°01'37.14	*Ilex* sp.	58	Coccidae	*Aonidomytilus* sp.	-	MH455968
Metaphycus stanleyi	23876	06/05/2015	V	Casablanca	33°27'25.54	71°23'40.84	*Schinus latifolius*	241	Coccidae	*Saissetia oleae*	-	MH456125
Metaphycus stanleyi	23877	06/05/2015	V	Cartagena	33°28'58.44	71°25'10.95	*Schinus latifolius*	218	Coccidae	*Saissetia oleae*	-	MH456126
Metaphycus stanleyi	24076	08/04/2015	V	La Cruz	32°51'15.99	71°11'04.56	*Persea americana*	214	Coccidae	*Saissetia oleae*	-	MH456094
Metaphycus stanleyi	24077	08/04/2015	V	La Cruz	32°51'15.99	71°11'04.56	*Persea americana*	214	Coccidae	*Saissetia oleae*	-	MH456095
Metaphycus stanleyi	24078	08/04/2015	V	La Cruz	32°51'15.99	71°11'04.56	*Persea americana*	214	Coccidae	*Saissetia oleae*	-	MH456096
Metaphycus stanleyi	24079	08/04/2015	V	La Cruz	32°51'15.99	71°11'04.56	*Persea americana*	214	Coccidae	*Saissetia oleae*	-	MH456097
Metaphycus stanleyi	24084	16/04/2015	V	Hijuelas	32°51'24.77	71°03'51.8	*Persea americana*	370	Coccidae	*Saissetia oleae*	-	MH456098
Metaphycus stanleyi	24085	16/04/2015	V	Hijuelas	32°51'24.77	71°03'51.8	*Persea americana*	370	Coccidae	*Saissetia oleae*	-	MH456099
Metaphycus stanleyi	24901	16/02/2016	IV	La Serena	29°55'08.61	71°14'33.31	*Citrus sinensis*	92	Coccidae	*Saissetia oleae*	-	MH456102

Species name	Molecular Code	Sample Date	Region	Municipality	Latitud	Longitud	Host plant	Altitude	Family Host	Species Host	GenBank Access COI	GenBank Access 28S
Metaphycus stanleyi	24916	16/02/2016	IV	La Serena	29°55'08.61	71°14'33.31	*Citrus sinensis*	92	Coccidae	*Saissetia oleae*	-	MH456104
Metaphycus stanleyi	24965	-	-	-	-	-	-	-	-	-	-	MH456107
Metaphycus stanleyi	24986	16/02/2016	IV	Vicuña	30°02'08.3	70°41'48.43	*Vitis vignifera*	632	Coccidae	*Coccus hesperidum*	-	MH456111
Metaphycus stanleyi	25002	16/02/2016	IV	Vicuña	30°02'08.3	70°41'48.43	*Vitis vignifera*	632	Coccidae	*Coccus hesperidum*	-	MH456113
Metaphycus stanleyi	25016	16/02/2016	IV	Vicuña	30°02'08.3	70°41'48.43	*Vitis vignifera*	632	Coccidae	*Coccus hesperidum*	-	MH456114
Metaphycus stanleyi	23689	02/06/2015	IV	Monte Patria	30°43'06.29	70°52'32.47	*Citrus* sp.	505	Coccidae	*Coccus hesperidum*	MH456614	MH456080
Metaphycus stanleyi	23690	02/06/2015	IV	Monte Patria	30°43'06.29	70°52'32.47	*Citrus* sp.	505	Coccidae	*Coccus hesperidum*	MH456615	MH456081
Metaphycus stanleyi	23771	06/05/2015	V	Casablanca	33°27'25.54	71°23'40.84	*Schinus latifolius*	241	Coccidae	*Saissetia oleae*	MH456618	MH456082
Metaphycus stanleyi	23776	01/04/2015	V	Cabildo	32°26'22.3	71°05'49.72	*Persea americana*	173	Diaspididae	*Saissetia oleae*	MH456619	MH456083
Metaphycus stanleyi	23837	14/05/2015	Met	La Pintana	33°34'07.93	70°38'03.37	*Olea europaea*	623	Coccidae	*Saissetia oleae*	MH456621	MH456123
Metaphycus stanleyi	23838	14/05/2015	Met	La Pintana	33°34'07.93	70°38'03.37	*Olea europaea*	623	Coccidae	*Saissetia oleae*	MH456622	MH456122
Metaphycus stanleyi	23839	14/05/2015	Met	La Pintana	33°34'07.93	70°38'03.37	*Olea europaea*	623	Coccidae	*Saissetia oleae*	MH456623	MH456124
Metaphycus stanleyi	25031	05/01/2016	Met	Paine	33°51'19.8	70°45'21.38	*Hedera helix*	378	Diaspididae	*Aonidiella ensifera*	-	MH456115
Metaphycus stanleyi	25032	26/11/2015	XV	Arica	18°34'57.31	69°57'09.14	*Olea europaea*	834	Coccidae	*Saissetia coffeae*	-	MH456116
Metaphycus stanleyi	23840	14/05/2015	Met	La Pintana	33°34'07.93	70°38'03.37	*Olea europaea*	623	Coccidae	*Saissetia oleae*	MH456624	MH456085
Metaphycus stanleyi	23908	08/04/2015	V	La Cruz	32°51'29.52	71°11'19.04	*Persea americana*	186	Coccidae	*Protopulvinaria pyriformis*	MH456625	MH456086
Metaphycus stanleyi	23929	01/04/2015	V	Cabildo	32°26'33.36	71°06'03.3	*Persea americana*	172	Coccidae	*Saissetia oleae*	MH456626	MH456079
Metaphycus stanleyi	23930	01/04/2015	V	Cabildo	32°26'33.36	71°06'03.3	*Persea americana*	172	Coccidae	*Saissetia oleae*	MH456627	MH456087
Metaphycus stanleyi	23718	02/06/2015	IV	Monte Patria	30°43'06.29	70°52'32.47	*Citrus* sp.	505	Coccidae	*Coccus hesperidum*	MH456616	-
Metaphycus stanleyi	23719	02/06/2015	IV	Monte Patria	30°43'06.29	70°52'32.47	*Citrus* sp.	505	Coccidae	*Coccus hesperidum*	MH456617	-
Metaphycus stanleyi	23973	25/05/2015	IV	Coquimbo	29°57'20.09	71°20'10.99	*Olea europaea*	6	Coccidae	*Saissetia coffeae*	MH456632	-
Metaphycus stanleyi	23958	01/04/2015	V	Cabildo	32°26'40.34	71°06'08.74	*Persea americana*	170	Diaspididae	*Hemiberlesia lataniae*	MH456628	MH456088
Metaphycus stanleyi	23967	24/05/2015	IV	Coquimbo	29°57'20.09	71°20'10.99	*Olea europaea*	6	Coccidae	*Saissetia coffeae*	MH456629	MH456089
Metaphycus stanleyi	23968	24/05/2015	IV	Coquimbo	29°57'20.09	71°20'10.99	*Olea europaea*	6	Coccidae	*Saissetia coffeae*	MH456630	MH456090
Metaphycus stanleyi	23972	25/05/2015	IV	Coquimbo	29°57'20.09	71°20'10.99	*Olea europaea*	6	Coccidae	*Saissetia coffeae*	MH456631	MH456091
Metaphycus stanleyi	24016	08/04/2015	V	La Cruz	32°51'20.09	71°11'26.48	*Olea europaea*	176	Coccidae	*Saissetia oleae*	MH456633	MH456092
Metaphycus stanleyi	24017	08/04/2015	V	La Cruz	32°51'20.09	71°11'26.48	*Olea europaea*	176	Coccidae	*Saissetia oleae*	MH456634	MH456093
Metaphycus stanleyi	24155	28/07/2015	V	Quillota	32°56'12.79	71°18'30.08	-	75	-	-	MH456635	MH456375
Metaphycus stanleyi	24156	28/07/2015	V	Quillota	32°56'12.79	71°18'30.08	-	75	-	-	MH456636	MH456376
Metaphycus stanleyi	24889	16/02/2016	IV	La Serena	29°55'08.61	71°14'33.31	*Citrus sinensis*	92	Coccidae	*Saissetia oleae*	MH456637	MH456100
Metaphycus stanleyi	24890	16/02/2016	IV	La Serena	29°55'08.61	71°14'33.31	*Citrus sinensis*	92	Coccidae	*Saissetia oleae*	MH456638	MH456101
Metaphycus stanleyi	24902	16/02/2016	IV	La Serena	29°55'08.61	71°14'33.31	*Citrus sinensis*	92	Coccidae	*Saissetia oleae*	MH456639	MH456103
Metaphycus stanleyi	24927	16/02/2016	IV	La Serena	29°55'08.61	71°14'33.31	*Citrus sinensis*	92	Coccidae	*Saissetia oleae*	MH456640	MH456105
Metaphycus stanleyi	24928	16/02/2016	IV	La Serena	29°55'08.61	71°14'33.31	*Citrus sinensis*	92	Coccidae	*Saissetia oleae*	MH456641	MH456106
Metaphycus stanleyi	24967	17/02/2016	IV	La Serena	29°56'22.49	71°08'50.13	*Olea europaea*	120	Diaspididae	*Saissetia coffeae*	MH456642	MH456108
Metaphycus stanleyi	24968	16/02/2016	IV	La Serena	29°55'08.61	71°14'33.31	*Hedera helix*	92	Coccidae	*Protopulvinaria pyriformis*	MH456643	MH456109
Metaphycus stanleyi	24985	17/02/2016	IV	La Serena	29°56'22.49	71°08'50.13	*Olea europaea*	120	Coccidae	*Saissetia coffeae*	MH456644	MH456110
Metaphycus stanleyi	25001	05/01/2016	VI	Chimbarongo	34°38'33.1	70°59'20.22	*Hedera helix*	374	Diaspididae	-	MH456645	MH456112
Metaphycus stanleyi	25110	16/12/2015	V	Quillota	32°56'06.20	71°15'28	*Citrus limon*	230	Diaspididae	*Diaspidiotus perniciosus*	-	MH456117
Metaphycus stanleyi	25136	18/12/2015	V	Quillota	32°51'47.44	71°11'02.85	*Persea americana*	201	Coccidae	*Protopulvinaria pyriformis*	-	MH456118
Metaphycus stanleyi	25137	18/12/2015	V	Quillota	32°51'47.44	71°11'02.85	*Persea americana*	201	Coccidae	*Protopulvinaria pyriformis*	-	MH456119
Metaphycus stanleyi	25147	18/12/2015	V	Quillota	32°51'47.44	71°11'02.85	*Persea americana*	201	Coccidae	*Protopulvinaria pyriformis*	-	MH456120
Metaphycus stanleyi	25148	18/12/2015	V	Quillota	32°51'47.44	71°11'02.85	*Persea americana*	201	Coccidae	*Protopulvinaria pyriformis*	-	MH456121
NA	27828	05/01/2016	VI	Chimbarongo	34°38'33.1	70°59'20.22	*Hedera helix*	374	Diaspididae	-	MH456613	-
NA	23707	28/05/2015	VII	Parral	36°08'48.21	71°49'15.61	*Aristotelia chilensis*	184	Coccidae	*Parthenolecanium corni*	MH456696	-
Pteromalidae	23937	17/04/2015	V	Quillota	32°50'54.81	71°13'57.89	*Citrus aurantifolia*	158	Diaspididae	*Lepidosaphes beckii*	-	MH455924
Pteromalidae	27827	05/01/2016	VI	Chimbarongo	34°38'33.1	70°59'20.22	*Hedera helix*	374	Diaspididae	-	MH456612	MH455925
Scutellista coerulea	23754	01/06/2015	IV	Ovalle	30°32'16.49	71°08'40.31	*Annona cherimola*	286	Coccidae	*Parasaissetia nigra*	-	MH455876
Scutellista coerulea	23831	21/05/2015	IV	La Serena	29°56'22.33	71°08'49.74	*Olea europaea*	120	Coccidae	*Saissetia coffeae*	-	MH455906
Scutellista coerulea	23933	01/04/2015	V	Cabildo	32°26'22.3	71°05'49.72	*Persea americana*	173	Coccidae	*Saissetia oleae*	-	MH455877
Scutellista coerulea	24011	26/03/2015	Met	San Bernando	33°40'09.98	70°43'20.9	*Nerium oleander*	544	Diaspididae	*Saissetia oleae*	-	MH455879
Scutellista coerulea	24024	26/03/2015	VI	San Fernando	34°30'26.88	70°55'20.31	*Schinus latifolius*	313	Coccidae	*Saissetia oleae*	-	MH455880
Scutellista coerulea	24036	08/04/2015	V	La Cruz	32°51'20.09	71°11'26.48	*Olea europaea*	176	Coccidae	*Saissetia oleae*	-	MH455881
Scutellista coerulea	24098	27/02/2015	V	Hijuelas	32°51'27.1	71°03'59.58	*Persea americana*	351	Coccidae	*Saissetia oleae*	-	MH455882

Species name	Molecular Code	Sample Date	Region	Municipality	Latitud	Longitud	Host plant	Altitude	Family Host	Species Host	GenBank Access COI	GenBank Access 28S
Scutellista coerulea	24105	27/02/2015	V	Hijuelas	32°51'27.1	71°03'59.58	*Persea americana*	351	Coccidae	*Saissetia oleae*	-	MH455883
Scutellista coerulea	24775	08/10/2015	VI	Rengo	34°21'22.75	70°50'45.48	*Nerium oleander*	338	Coccidae	*Saissetia oleae*	-	MH455884
Scutellista coerulea	24776	08/10/2015	VI	Rengo	34°21'22.75	70°50'45.48	*Nerium oleander*	338	Coccidae	*Saissetia oleae*	-	MH455885
Scutellista coerulea	24778	21/09/2015	V	Catemu	32°47'28.81	70°52'11.37	*Nerium oleander*	499	Coccidae	*Saissetia oleae*	-	MH455886
Scutellista coerulea	24779	21/09/2015	V	Catemu	32°47'28.81	70°52'11.37	*Nerium oleander*	499	Coccidae	*Saissetia oleae*	-	MH455887
Scutellista coerulea	24909	06/01/2016	V	Putaendo	32°37'21.76	70°43'00.69	*Citrus sinensis*	831	Coccidae	*Saissetia oleae*	-	MH455888
Scutellista coerulea	24935	06/01/2016	V	Putaendo	32°37'21.76	70°43'00.69	*Citrus sinensis*	831	Coccidae	*Saissetia oleae*	-	MH455890
Scutellista coerulea	24957	05/01/2016	Met	Paine	33°51'05.26	70°45'50.46	*Olea europaea*	380	Coccidae	*Saissetia oleae*	-	MH455891
Scutellista coerulea	24970	17/02/2016	IV	La Serena	29°56'22.49	71°08'50.13	*Olea europaea*	120	Coccidae	*Saissetia coffeae*	-	MH455892
Scutellista coerulea	24984	17/02/2016	IV	La Serena	29°56'22.49	71°08'50.13	*Olea europaea*	120	Coccidae	*Saissetia coffeae*	-	MH455893
Scutellista coerulea	25025	16/02/2016	III	Vallenar	28°34'51.69	70°47'49.57	*Citrus sinensis*	470	Diaspididae	*Lepidosaphes beckii*	-	MH455895
Scutellista coerulea	25026	26/11/2015	XV	Arica	18°34'57.31	69°57'09.14	*Olea europaea*	834	Diaspididae	*Hemiberlesia palmae*	-	MH455896
Scutellista coerulea	24010	26/03/2015	Met	San Bernando	33°40'09.98	70°43'20.9	*Nerium oleander*	544	Diaspididae	*Saissetia oleae*	MH456766	MH455878
Scutellista coerulea	24037	08/04/2015	V	La Cruz	32°51'20.09	71°11'26.48	*Olea europaea*	176	Coccidae	*Saissetia oleae*	MH456768	MH455905
Scutellista coerulea	24023	26/03/2015	VI	San Fernando	34°30'26.88	70°55'20.31	*Schinus latifolius*	313	Coccidae	*Saissetia oleae*	MH456767	-
Scutellista coerulea	27837	06/01/2016	V	Catemu	32°47'28.56	70°52'11.09	*Nerium oleander*	499	Coccidae	-	MH456770	-
Scutellista coerulea	24934	06/01/2016	V	Putaendo	32°37'21.76	70°43'00.69	*Citrus sinensis*	831	Coccidae	*Saissetia oleae*	MH456769	MH455889
Scutellista coerulea	27880	26/11/2015	XV	Arica	18°34'57.31	69°57'09.14	*Olea europaea*	834	Coccidae	*Saissetia coffeae*	MH456771	MH455900
Scutellista coerulea	27944	07/01/2016	VII	Curico	34°59'01.09	71°13'17.73	*Citrus sinensis*	223	Coccidae	*Saissetia oleae*	MH456772	MH455903
Scutellista coerulea	27814	05/01/2016	VII	Parral	36°08'58.12	71°49'15.83	*Nerium oleander*	182	Coccidae	*Saissetia oleae*	-	MH455897
Scutellista coerulea	27871	05/01/2016	Met	Paine	33°51'17.41	70°45'49.47	*Citrus limon*	375	Coccidae	*Saissetia oleae*	-	MH455898
Scutellista coerulea	27879	26/11/2015	XV	Arica	18°34'57.31	69°57'09.14	*Olea europaea*	834	Coccidae	*Saissetia coffeae*	-	MH455899
Scutellista coerulea	27937	18/12/2015	V	Quillota	32°51'54.4	71°11'10.98	*Schinus latifolius*	185	Coccidae	*Saissetia oleae*	-	MH455901
Scutellista coerulea	27943	05/01/2016	VII	Linares	35°50'32.02	71°37'01.25	*Nerium oleander*	156	Coccidae	*Saissetia oleae*	-	MH455902
Scutellista coerulea	27945	07/01/2016	VII	Curico	34°59'01.09	71°13'17.73	*Citrus sinensis*	223	Coccidae	*Saissetia oleae*	-	MH455904
Signiphora bifasciata	23845	14/05/2015	Met	La Pintana	33°34'07.93	70°38'03.37	*Olea europaea*	623	Coccidae	*Saissetia oleae*	-	MH456227
Signiphora bifasciata	23846	14/05/2015	Met	La Pintana	33°34'07.93	70°38'03.37	*Olea europaea*	623	Coccidae	*Saissetia oleae*	-	MH456228
Signiphora bifasciata	23865	19/03/2015	VI	Pelequen	34°26'05.43	70°55'22.88	Unknown	278	Coccidae	*Saissetia oleae*	-	MH456225
Signiphora bifasciata	23866	19/03/2015	VI	Pelequen	34°26'05.43	70°55'22.88	Unknown	278	Coccidae	*Saissetia oleae*	-	MH456223
Signiphora bifasciata	23868	19/03/2015	VI	Pelequen	34°26'05.43	70°55'22.88	Unknown	278	Coccidae	*Saissetia oleae*	-	MH456224
Signiphora bifasciata	24001	01/06/2015	IV	Ovalle	30°32'16.49	71°08'40.31	*Annona cherimola*	286	Coccidae	*Parasaissetia nigra*	-	MH456231
Signiphora bifasciata	24141	16/04/2015	V	Hijuelas	32°51'55.35	71°04'01.65	*Schinus latifolius*	471	Coccidae	*Saissetia oleae*	-	MH456235
Signiphora bifasciata	24142	16/04/2015	V	Hijuelas	32°51'55.35	71°04'01.65	*Schinus latifolius*	471	Coccidae	*Saissetia oleae*	-	MH456236
Signiphora bifasciata	25107	16/12/2015	V	Quillota	32°56'06.20	71°15'28	*Citrus limon*	230	Coccidae	*Ceroplastes sinensis*	-	MH456237
Signiphora bifasciata	23768	01/06/2015	IV	Ovalle	30°32'16.49	71°08'40.31	*Annona cherimola*	286	Coccidae	*Parasaissetia nigra*	MH456703	MH456242
Signiphora bifasciata	23769	01/06/2015	IV	Ovalle	30°32'16.49	71°08'40.31	*Annona cherimola*	286	Coccidae	*Parasaissetia nigra*	MH456704	MH456226
Signiphora bifasciata	23909	08/04/2015	V	La Cruz	32°51'15.07	71°11'59.30	*Cryptocarya alba*	146	Coccidae	*Protopulvinaria pyriformis*	MH456705	MH456229
Signiphora bifasciata	23910	08/04/2015	V	La Cruz	32°51'15.07	71°11'59.30	*Cryptocarya alba*	146	Coccidae	*Protopulvinaria pyriformis*	MH456706	MH456230
Signiphora bifasciata	25125	16/12/2015	V	Quillota	32°56'06.20	71°15'28	*Citrus limon*	230	Coccidae	*Ceroplastes sinensis*	-	MH456238
Signiphora bifasciata	24002	01/06/2015	IV	Ovalle	30°32'16.49	71°08'40.31	*Annona cherimola*	286	Coccidae	*Parasaissetia nigra*	MH456707	MH456232
Signiphora bifasciata	24040	08/04/2015	V	La Cruz	32°51'20.09	71°11'26.48	*Olea europaea*	176	Coccidae	*Saissetia oleae*	MH456708	MH456233
Signiphora bifasciata	24041	08/04/2015	V	La Cruz	32°51'20.09	71°11'26.48	*Olea europaea*	176	Coccidae	*Saissetia oleae*	MH456709	MH456234
Signiphora bifasciata	24153	18/08/2015	V	La Cruz	32°49'40.88	71°13'37.89	-	176	-	-	MH456710	MH456373
Signiphora bifasciata	27818	05/01/2016	Met	Paine	33°51'19.8	70°45'21.38	*Hedera helix*	378	Coccidae	*Protopulvinaria pyriformis*	MH456711	MH456240
Signiphora bifasciata	27866	05/01/2016	Met	Paine	33°51'17.41	70°45'49.47	*Citrus limon*	375	Coccidae	*Saissetia oleae*	MH456712	MH456241
Signiphora bifasciata	27816	05/01/2016	VII	Parral	36°08'58.12	71°49'15.83	*Nerium oleander*	182	Coccidae	*Saissetia oleae*	-	MH456239
Signiphora flavella	23918	16/04/2015	V	Hijuelas	32°51'47.44	71°03'39.48	*Persea americana*	506	Diaspididae	*Aspidiotus nerii*	-	MH456271
Signiphora flavella	24115	02/06/2015	IV	Monte Patria	30°43'49.4	70°52'53.19	*Persea americana*	565	Diaspididae	*Hemiberlesia lataniae*	-	MH456260
Signiphora flavella	24119	02/06/2015	IV	Monte Patria	30°51'04.56	70°46'14.47	*Persea americana*	715	Diaspididae	*Hemiberlesia lataniae*	-	MH456279
Signiphora flavella	23735	02/06/2015	IV	Ovalle	30°32'16.49	71°08'40.31	*Prunus persica*	286	Diaspididae	*Lepidosaphes ulmi*	MH456540	MH456280
Signiphora flavella	23747	02/06/2015	IV	Monte Patria	30°43'49.4	70°52'53.19	*Persea americana*	565	Diaspididae	*Hemiberlesia lataniae*	MH456541	MH456263
Signiphora flavella	23748	02/06/2015	IV	Monte Patria	30°43'49.4	70°52'53.19	*Persea americana*	565	Diaspididae	*Hemiberlesia lataniae*	MH456542	MH456264
Signiphora flavella	23749	02/06/2015	IV	Monte Patria	30°43'49.4	70°52'53.19	*Persea americana*	565	Diaspididae	*Hemiberlesia lataniae*	MH456543	MH456265
Signiphora flavella	23750	02/06/2015	IV	Monte Patria	30°43'49.4	70°52'53.19	*Persea americana*	565	Diaspididae	*Hemiberlesia lataniae*	MH456544	MH456266

Species name	Molecular Code	Sample Date	Region	Municipality	Latitud	Longitud	Host plant	Altitude	Family Host	Species Host	GenBank Access COI	GenBank Access 28S
Signiphora flavella	23752	02/06/2015	IV	Monte Patria	30°51'04.56	70°46'14.47	*Persea americana*	715	Diaspididae	*Lepidosaphes ulmi*	MH456545	MH456267
Signiphora flavella	23763	02/06/2015	IV	Monte Patria	30°51'04.56	70°46'14.47	*Persea americana*	715	Diaspididae	*Hemiberlesia lataniae*	MH456546	MH456268
Signiphora flavella	23764	02/06/2015	IV	Monte Patria	30°51'04.56	70°46'14.47	*Persea americana*	715	Diaspididae	*Hemiberlesia lataniae*	MH456547	MH456269
Signiphora flavella	23850	02/06/2015	IV	Monte Patria	30°43'49.4	70°52'53.19	*Persea americana*	565	Diaspididae	*Hemiberlesia lataniae*	MH456548	MH456262
Signiphora flavella	23851	02/06/2015	IV	Monte Patria	30°43'49.4	70°52'53.19	*Persea americana*	565	Diaspididae	*Hemiberlesia lataniae*	MH456549	MH456270
Signiphora flavella	23852	02/06/2015	IV	Monte Patria	30°43'49.4	70°52'53.19	*Persea americana*	565	Diaspididae	*Hemiberlesia lataniae*	MH456550	MH456261
Signiphora flavella	23964	01/04/2015	V	Cabildo	32°26'46.47	71°05'35.7	*Persea americana*	159	Diaspididae	-	MH456552	-
Signiphora flavella	23951	01/04/2015	V	Cabildo	32°26'33.36	71°06'03.3	*Persea americana*	172	Coccidae	*Saissetia oleae*	MH456551	MH456272
Signiphora flavella	24025	08/04/2015	V	La Cruz	32°51'19.59	71°11'16.13	*Persea americana*	215	Diaspididae	*Hemiberlesia lataniae*	MH456553	MH456273
Signiphora flavella	24026	08/04/2015	V	La Cruz	32°51'19.59	71°11'16.13	*Persea americana*	215	Diaspididae	*Hemiberlesia lataniae*	MH456554	MH456274
Signiphora flavella	24027	08/04/2015	V	La Cruz	32°51'19.59	71°11'16.13	*Persea americana*	215	Diaspididae	*Hemiberlesia lataniae*	MH456555	MH456275
Signiphora flavella	24056	02/04/2015	VII	Pencahue	35°26'16.16	71°49'12.11	*Olea europaea*	72	Diaspididae	*Aspidiotus nerii*	MH456557	MH456277
Signiphora flavella	24057	02/04/2015	VII	Pencahue	35°26'16.16	71°49'12.11	*Olea europaea*	72	Diaspididae	*Aspidiotus nerii*	MH456558	MH456278
Signiphora perpauca	24804	07/10/2015	VIII	San Carlos	36°26'03.47	71°57'45.46	*Ilex* sp.	184	Diaspididae	*Aspidiotus nerii*	-	MH456250
Signiphora perpauca	23855	28/05/2015	VIII	San Carlos	36°26'04.58	71°57'45.18	*Ilex* sp.	185	Diaspididae	*Aspidiotus nerii*	MH456745	MH456258
Signiphora perpauca	23856	28/05/2015	VIII	San Carlos	36°26'04.58	71°57'45.18	*Ilex* sp.	185	Diaspididae	*Aspidiotus nerii*	MH456746	MH456259
Signiphora perpauca	24943	16/02/2016	III	Huasco	28°28'59.11	71°09'24.76	*Nerium oleander*	43	Diaspididae	*Aspidiotus nerii*	MH456748	-
Signiphora perpauca	24805	07/10/2015	VIII	San Carlos	36°26'03.47	71°57'45.46	*Ilex* sp.	184	Diaspididae	*Aspidiotus nerii*	MH456747	MH456249
Signiphora sp.I	23765	02/06/2015	IV	Ovalle	30°32'16.49	71°08'40.31	*Citrus limon*	286	Diaspididae	*Lepidosaphes beckii*	MH456750	MH456312
Signiphora sp.I	23766	02/06/2015	IV	Ovalle	30°32'16.49	71°08'40.31	*Citrus limon*	286	Diaspididae	*Lepidosaphes beckii*	MH456751	MH456313
Signiphora sp.I	23883	02/06/2015	IV	Ovalle	30°32'16.49	71°08'40.31	*Citrus limon*	286	Diaspididae	*Lepidosaphes beckii*	MH456749	MH456314
Signiphora sp.II	23725	02/06/2015	IV	Monte Patria	30°53'41.72	70°39'28.89	*Olea europaea*	1278	Diaspididae	-	-	MH456245
Signiphora sp.II	23740	02/06/2015	IV	Monte Patria	30°51'04.56	70°46'14.47	*Persea americana*	715	Diaspididae	*Hemiberlesia lataniae*	-	MH456246
Signiphora sp.II	23998	02/06/2015	IV	Monte Patria	30°53'41.72	70°39'28.89	*Olea europaea*	1278	Diaspididae	-	-	MH456247
Signiphora sp.II	24915	16/02/2016	III	Vallenar	28°34'51.69	70°47'49.57	*Citrus sinensis*	470	Diaspididae	*Lepidosaphes beckii*	-	MH456248
Signiphora sp.II	23941	17/04/2015	V	Quillota	32°50'54.81	71°13'57.89	*Citrus aurantifolia*	158	Diaspididae	*Lepidosaphes beckii*	MH456755	MH456243
Signiphora sp.II	23942	17/04/2015	V	Quillota	32°50'54.81	71°13'57.89	*Citrus aurantifolia*	158	Diaspididae	*Lepidosaphes beckii*	MH456754	MH456244
Signiphora sp.III	23794	26/03/2015	VII	Sagrada Familia	35°02'04.84	71°18'23.49	*Aristotelia chilensis*	200	Diaspididae	*Hemiberlesia rapax*	-	MH456293
Signiphora sp.III	23811	26/03/2015	VII	Sagrada Familia	35°02'04.84	71°18'23.49	*Aristotelia chilensis*	200	Diaspididae	*Hemiberlesia rapax*	-	MH456296
Signiphora sp.III	23812	26/03/2015	VII	Sagrada Familia	35°02'04.84	71°18'23.49	*Aristotelia chilensis*	200	Diaspididae	*Hemiberlesia rapax*	-	MH456310
Signiphora sp.III	23814	26/03/2015	VII	Sagrada Familia	35°02'04.84	71°18'23.49	*Aristotelia chilensis*	200	Diaspididae	*Hemiberlesia rapax*	-	MH456283
Signiphora sp.III	23860	28/05/2015	VIII	San Carlos	36°26'04.58	71°57'45.18	*Ilex* sp.	185	Diaspididae	*Aspidiotus nerii*	-	MH456311
Signiphora sp.III	23892	06/05/2015	V	Cartagena	33°28'58.44	71°25'10.95	*Schinus latifolius*	218	Coccidae	*Saissetia oleae*	-	MH456282
Signiphora sp.III	23893	06/05/2015	V	Cartagena	33°28'58.44	71°25'10.95	*Schinus latifolius*	218	Coccidae	*Saissetia oleae*	-	MH456284
Signiphora sp.III	23979	02/06/2015	IV	Monte Patria	30°43'14.76	70°52'16.89	*Persea americana*	563	Diaspididae	*Hemiberlesia lataniae*	-	MH456285
Signiphora sp.III	24012	01/04/2015	V	Cabildo	32°26'18.05	71°06'12.46	*Persea americana*	240	Diaspididae	*Saissetia oleae*	-	MH456286
Signiphora sp.III	24013	01/04/2015	V	Cabildo	32°26'18.05	71°06'12.46	*Persea americana*	240	Diaspididae	*Saissetia oleae*	-	MH456287
Signiphora sp.III	24111	02/06/2015	IV	Monte Patria	30°43'14.76	70°52'16.89	*Persea americana*	563	Diaspididae	*Hemiberlesia lataniae*	-	MH456281
Signiphora sp.III	24113	02/06/2015	IV	Monte Patria	30°43'14.76	70°52'16.89	*Persea americana*	563	Diaspididae	*Hemiberlesia lataniae*	-	MH456288
Signiphora sp.III	24788	09/09/2015	V	Quillota	32°51'45.95	71°11'23.69	*Persea americana*	172	Diaspididae	*Hemiberlesia lataniae*	-	MH456289
Signiphora sp.III	24789	09/09/2015	V	Quillota	32°51'45.95	71°11'23.69	*Persea americana*	172	Diaspididae	*Hemiberlesia lataniae*	-	MH456290
Signiphora sp.III	24855	21/09/2015	V	Cabildo	32°26'24.40	71°06'12.48	*Persea americana*	216	Diaspididae	*Hemiberlesia lataniae*	-	MH456291
Signiphora sp.III	24856	21/09/2015	V	Cabildo	32°26'24.40	71°06'12.48	*Persea americana*	216	Diaspididae	*Hemiberlesia lataniae*	-	MH456292
Signiphora sp.III	27794	05/01/2016	VIII	San Carlos	36°26'03.53	71°57'45.15	*Ilex* sp.	184	Diaspididae	*Aspidiotus nerii*	-	MH456307
Signiphora sp.III	23736	28/05/2015	VIII	San Carlos	36°26'04.58	71°57'45.18	*Aristotelia chilensis*	185	Diaspididae	*Hemiberlesia rapax*	MH456675	MH456309
Signiphora sp.III	23737	28/05/2015	VIII	San Carlos	36°26'04.58	71°57'45.18	*Aristotelia chilensis*	185	Diaspididae	*Hemiberlesia rapax*	MH456676	MH456295
Signiphora sp.III	23895	06/05/2015	V	Cartagena	33°28'58.44	71°25'10.95	*Schinus latifolius*	218	Coccidae	*Saissetia oleae*	MH456677	MH456297
Signiphora sp.III	23896	06/05/2015	V	Cartagena	33°28'58.44	71°25'10.95	*Schinus latifolius*	218	Coccidae	*Saissetia oleae*	MH456678	MH456298
Signiphora sp.III	23988	28/05/2015	VIII	San Carlos	36°26'04.58	71°57'45.18	*Aristotelia chilensis*	185	Diaspididae	*Hemiberlesia rapax*	MH456679	MH456299
Signiphora sp.III	23989	28/05/2015	VIII	San Carlos	36°26'04.58	71°57'45.18	*Aristotelia chilensis*	185	Diaspididae	*Hemiberlesia rapax*	MH456680	MH456300
Signiphora sp.III	23990	28/05/2015	VIII	San Carlos	36°26'04.58	71°57'45.18	*Aristotelia chilensis*	185	Diaspididae	*Hemiberlesia rapax*	MH456681	MH456301
Signiphora sp.III	23991	21/05/2015	IV	La Serena	29°54'58.14	71°14'43.09	*Hedera helix*	47	Coccidae	*Protopulvinaria pyriformis*	MH456758	MH456294
Signiphora sp.III	23698	28/05/2015	VIII	San Carlos	36°26'04.58	71°57'45.18	*Aristotelia chilensis*	185	Diaspididae	*Hemiberlesia rapax*	MH456674	-
Signiphora sp.III	24058	24/05/2015	IV	Coquimbo	29°57'20.09	71°20'10.99	*Olea europaea*	6	Diaspididae	Diaspididae	MH456682	MH456302

Species name	Molecular Code	Sample Date	Region	Municipality	Latitud	Longitud	Host plant	Altitude	Family Host	Species Host	GenBank Access COI	GenBank Access 28S
Signiphora sp.III	24999	16/02/2016	III	Huasco	28°28'53.84	71°09'28.44	*Olea europaea*	37	Diaspididae	-	MH456683	MH456303
Signiphora sp.III	25000	05/01/2016	VI	Chimbarongo	34°38'33.1	70°59'20.22	*Hedera helix*	374	Diaspididae	-	MH456684	MH456304
Signiphora sp.III	25027	26/11/2015	XV	Arica	18°34'57.31	69°57'09.14	*Olea europaea*	834	Diaspididae	*Hemiberlesia palmae*	MH456685	MH456305
Signiphora sp.III	25028	26/11/2015	XV	Arica	18°34'57.31	69°57'09.14	*Olea europaea*	834	Diaspididae	*Hemiberlesia palmae*	MH456686	MH456306
Signiphora sp.III	27932	26/11/2015	X	Osorno	40°35'55.68	72°58'52.36	*Hedera helix*	91	Coccidae	*Pulvinariella mesembryanthemi*	MH456687	MH456308
Signiphora sp.IV	24014	08/04/2015	V	La Cruz	32°51'34.36	71°11'09.54	*Persea americana*	191	Diaspididae	*Hemiberlesia lataniae*	-	MH456254
Signiphora sp.IV	25086	17/11/2015	XIV	Valdivia	39°48'22.55	73°15'03.7	Unknown	18	Diaspididae	*Aonidomytilus sp.*	-	MH456253
Signiphora sp.IV	24009	08/04/2015	V	La Cruz	32°51'34.36	71°11'09.54	*Persea americana*	191	Diaspididae	*Hemiberlesia lataniae*	MH456788	MH456256
Signiphora sp.IV	25141	18/12/2015	V	Quillota	32°51'54.4	71°11'10.98	*Schinus latifolius*	185	Diaspididae	*Saissetia oleae*	MH456789	MH456252
Signiphora sp.IV	25142	18/12/2015	V	Quillota	32°51'54.4	71°11'10.98	*Schinus latifolius*	185	Diaspididae	*Saissetia oleae*	MH456785	MH456255
Signiphora sp.IV	25149	18/12/2015	V	Quillota	32°51'46.01	71°11'23.94	*Persea americana*	172	Diaspididae	*Hemiberlesia lataniae*	MH456787	MH456257
Signiphora sp.IV	25153	18/12/2015	V	Quillota	32°51'54.4	71°11'10.98	*Schinus latifolius*	185	Diaspididae	*Saissetia oleae*	MH456786	MH456251
Tetrastichinae sp.	23756	28/05/2015	VIII	San Carlos	36°25'58.64	71°57'42.88	*Maytenus boaria*	181	Coccidae	*Saissetia oleae*	MH456698	MH455620
Tetrastichinae sp.	24863	-	-	-	-	-	-	-	-	-	MH456699	MH455624
Tetrastichinae sp.	24864	-	-	-	-	-	-	-	-	-	MH456700	MH455621
Tetrastichinae sp.	25021	05/01/2016	VIII	San Carlos	36°26'13.64	71°57'45.56	*Olea europaea*	179	Coccidae	*Saissetia oleae*	MH456701	MH455622
Tetrastichinae sp.	25022	16/02/2016	III	Vallenar	28°34'43.53	70°47'42.21	*Olea europaea*	452	Coccidae	*Saissetia coffeae*	MH456702	MH455623
