# Supplementary figures and images for: Natural enemies of armored scales (Hemiptera: Diaspididae) and soft scales (Hemiptera: Coccidae) in Chile: Molecular and morphological identification
Source: PLoS One. 2019 Mar 18;14(3):e0205475. doi: 10.1371/journal.pone.0205475 (PMC6422274; doi:10.1371/journal.pone.0205475)

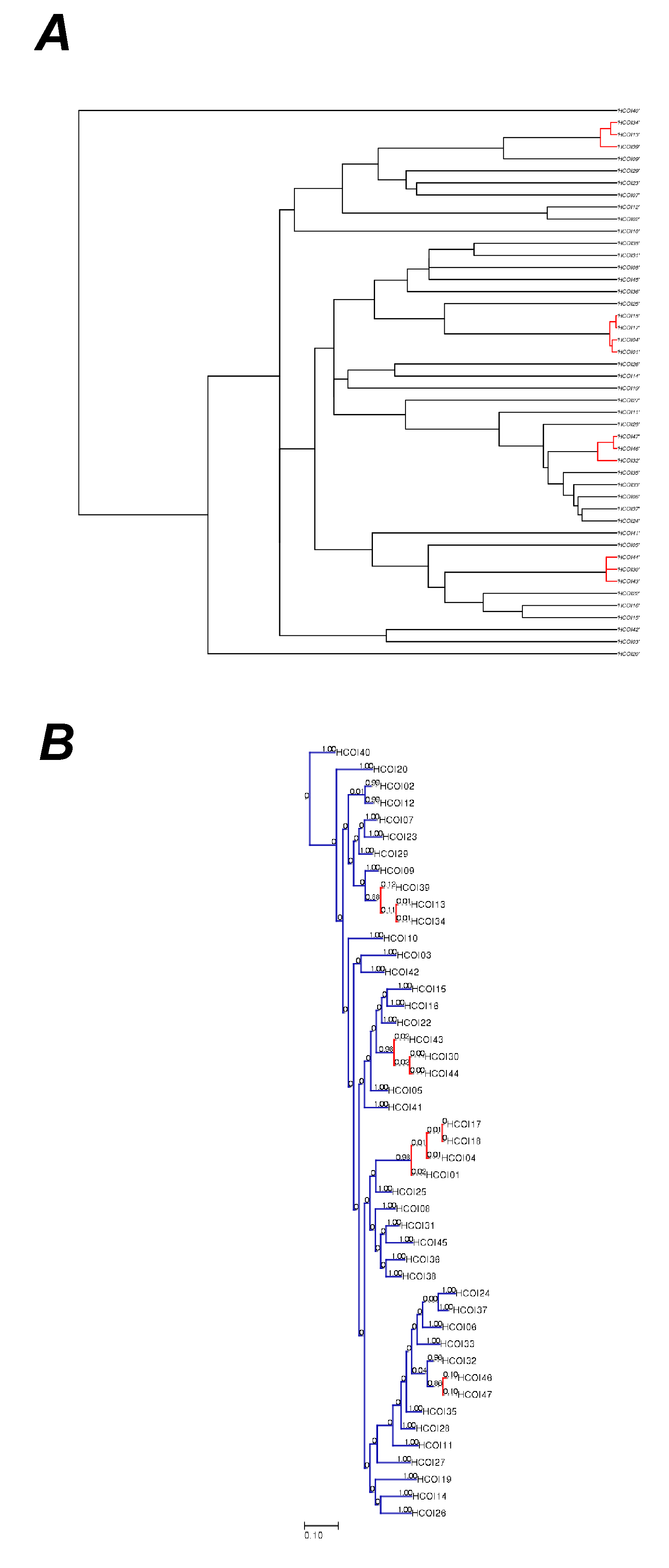

Supplement: S1 Fig — (PNG) [file pone.0205475.s002.png]
